# Supplementary material for: Unusual layer-by-layer growth of epitaxial oxide islands during Cu oxidation
Source: Nat Commun. 2021 May 13;12:2781. doi: 10.1038/s41467-021-23043-w (PMC8119701; doi:10.1038/s41467-021-23043-w)
Supplement: Supplementary file 1 — Supplementary Information [file 41467_2021_23043_MOESM1_ESM.pdf]

Supplementary Information for

Unusual layer-by-layer growth of epitaxial oxide islands during Cu oxidation

Meng Li[1], Matthew T. Curnan[1], Michael A. Gresh-Sill[1], Stephen D. House[1,3], Wissam A. Saidi[2] and Judith C. Yang[1,3,4]

[1]*Department of Chemical and Petroleum Engineering, University of Pittsburgh, Pittsburgh, PA (USA)*

[2]*Department of Mechanical Engineering & Materials Science, University of Pittsburgh, Pittsburgh, PA (USA)*

[3]*Environmental TEM Catalysis Consortium (ECC), University of Pittsburgh, Pittsburgh, PA (USA)*

[4]*Department of Physics and Astronomy, University of Pittsburgh, Pittsburgh, PA (USA)*

Correspondence to: alsaidi@pitt.edu, judyyang@pitt.edu

This PDF file includes:

Supplementary Methods
Supplementary Notes 1-8
Supplementary Figures 1-31
Supplementary Tables 1-7

Other Supplementary Materials for this manuscript include the following:

Supplementary Movies 1-5
Legends for Supplementary Movies 1-5

Table of Contents

Supplementary Methods **4**

Supplementary Figure 1 | TEM images of the as-prepared, pretreated, and oxidized Cu film sample. 7

Supplementary Note 1: Additional experimental results for layer-by-layer Cu_2O growth along $Cu_2O(110)$ **8**

Supplementary Figure 2 | 3D morphology of the Cu_2O islands. 9

Supplementary Figure 3 | Layer-by-layer growth of Cu_2O on Cu(100) and (110) surfaces..... 10

Supplementary Figure 4 | Cu_2O island growth on Cu(001) film surface 11

Supplementary Figure 5 | Layer-by-layer growth of Cu_2O in later oxidation stage. 12

Supplementary Figure 6 | Random nucleation sites of each new Cu_2O monolayer. 12

Supplementary Figure 7 | Oscillatory growth of $Cu_2O(110)$ monolayer. 13

Supplementary Figure 8 | Adjustment of the top of the island during oxidation. 13

Supplementary Figure 9 | Reconstructed Cu(100) surface during oxidation. 14

Supplementary Figure 10 | Cu_2O islands formed via interface Cu diffusion. 14

Supplementary Note 2: E-beam effect **15**

Supplementary Figure 11 | Formation of surface reconstruction on Cu when O_2 was injected. 16

Supplementary Note 3: Data analysis **17**

Supplementary Figure 12 | Extracted growth profile of each layer. 18

Supplementary Figure 13 | Additional growth rate analysis results. 19

Supplementary Figure 14 | Cubic fitting result of each layer using function $l^3=At$. 20

Supplementary Note 4: Statistical analysis of the growth rate **21**

Supplementary Figure 15 | Initial times and oxide layer range guesses over the tested structural breaks. 21

Supplementary Table 1 | Breakpoint guesses for intralayer oxide nucleation to growth transitions (T), interlayer nucleation (N) of new oxide layers, and concerted diffusion through layers or change in Cu position (P). 22

Supplementary Figure 16 | Statistically verified structural breakpoints. 23

Supplementary Table 2 | Numerical summary of univariate screening structural break analysis. 24

Supplementary Figure 17 | Q-Q plots of multivariate regression standardized residuals.......... 28

Supplementary Table 3 | Multivariate analysis summary of P6 and N4 structural breaks 29

Supplementary Figure 18 | Overlapping probability sample spaces of each layer in multivariate analysis. 30

Supplementary Note 5: DFT results on gas/solid interfacial energies **33**

Supplementary Figure 19 | γ of Cu-terminated $Cu_2O(100)$ surface with increasing Cu_xO_y surface units. 33

Supplementary Figure 20 | γ of O-terminated $Cu_2O(100)$ surface with increasing Cu_xO_y surface units. 34

Supplementary Figure 21 | γ of Cu-terminated $Cu_2O(110)$ surface with increasing Cu_xO_y surface units 34

Supplementary Figure 22 | γ of Cu-O terminated $Cu_2O(110)$ surface with increasing Cu_xO_y surface units 35

Supplementary Figure 23 | Plot of the calculated γ with increasing Cu_xO_y surface units. 35

Supplementary Table 4 | Summary of interfacial energies of grown Cu_xO_y surface unit calculations described in Supplementary Figures 19-22. 36
Supplementary Note 6: DFT results for adsorption sites on Cu_2O surfaces during oxide growth 37
Supplementary Figure 24 | 1st-4th adatom adsorption states on Cu terminated $Cu_2O(110)$ surface. 37
Supplementary Figure 25 | 1st-5th adatom adsorption states on Cu-O terminated $Cu_2O(110)$ surface. 38
Supplementary Figure 26 | Most favorable gas-solid interface energies of intermediate structures observed while growing Cu_xO_y structure (*ii*, Figure 2) on $Cu_2O(110)$. 39
Supplementary Table 5 | Summary of relative per adatom surface adsorption energies described in Supplementary Figures 24-25. 40
Supplementary Note 7: DFT results on Cu and O diffusion on Cu_2O surfaces 41
Supplementary Figure 27 | Adatom diffusion events on Cu-terminated $Cu_2O(100)$ forming the 0.5 layer (*ii,* Figure 2) Cu_2O structure. 41
Supplementary Figure 28 | Adatom diffusion events on Cu-terminated $Cu_2O(100)$ forming the 1.0 layer (*iii,* Figure 2) Cu_2O structures. 42
Supplementary Figure 29 | Cu adatom diffusion events on Cu-O terminated $Cu_2O(110)$ forming the 0.5 layer (*ii,* Figure 2) Cu_2O structure. 42
Supplementary Figure 30 | Adatom diffusion events on Cu terminated $Cu_2O(110)$ to form the 0.5 layer (*ii,* Figure 2) Cu_2O structure. 43
Supplementary Figure 31 | Summary of most favorable Cu and O diffusion processes on Cu_2O surfaces during oxidation, indicating adsorption (E_{ads}) and diffusion (Δ) energies for each process. 45
Supplementary Table 6 | Summary of diffusion energetics of visualized structures. 46
Supplementary Note 8: Evaluating the effect of surface reconstruction on oxide growth .. 47
Supplementary Table 7| Summary of Cu and O diffusion barriers on different Cu and Cu_2O surface configurations 48
Supplementary References 49

Supplementary Methods

1. Cu thin film preparation

Single crystalline Cu(100) thin films with a nominal thickness of 60 nm were grown on NaCl(100) substrates using UHV *e*-beam evaporation (Pascal Technologies UHV Dual *e*-beam Evaporator) with a base pressure of 1×10^{-9} Torr. Cu pallet evaporation material with a purity of 99.999% purchased from Kurt J. Lesker Company was used for Cu film evaporation. The thickness of the films was calibrated by a Quartz Crystal Microbalance (QCM) near the sample area. The NaCl substrates were freshly cleaved along their (100) planes to obtain clean, flat surfaces before evaporation. Evaporation was carried out in a furnace at elevated temperature (300 °C), while evaporated films were kept at this temperature for at least 30 min before cooling them down for better film quality.

The TEM samples were prepared by transferring these thin films onto Cu mesh grids (Ted Pella) using the float-off method. The NaCl substrates with deposited Cu films on top were cut into ~2×2 mm squares and put in super-pure water to dissolve some NaCl and make the Cu films float. Subsequently, floating Cu films were then lifted out using Cu mesh grids. This float-off process was applied iteratively using new super-pure water several times to remove residual NaCl. The films were stored inside a vacuum chamber (Fisher Scientific) with a base pressure of 37.5 Torr before ETEM experiments to avoid oxidation. To further avoid sample oxidation, ETEM experiments were performed within 2 days of evaporation.

2. *In situ* ETEM experiment

A differentially pumped Environmental TEM (Hitachi H-9500 ETEM) located at Nanoscale Fabrication and Characterization Facility (NFCF) at the University of Pittsburgh was used for the *in situ* ETEM experiment. This ETEM is operated at 300 keV with a LaB_6 electron gun and a home-built gas delivery system of up to three gas lines. High purity (99.99%) O_2 and H_2 gas tanks are connected to two of the gas lines. This home-built gas delivery system is connected to the vacuum pumping system of the TEM, so that switching between different gas species can be quickly achieved within a few minutes. A Hitachi double tilt heating holder was used for the experiment.

Sample pretreatment. To study the initial oxide growth process, unoxidized clean metal surfaces are required. Although fresh evaporated samples were used to avoid oxidation, air exposure during sample transfer can still cause slight oxidation of the sample (Supplementary Figure 1(a)). Hence, pretreatment of the sample is carried out inside the ETEM to remove native oxides and generate fresh & clean Cu surfaces. During the pretreatment process, Cu thin film samples were annealed at 600 °C with ~1 mTorr flowing H_2 gas to reduce native oxides and remove other contaminants on metal surfaces, as well as further remove defects such as dislocations caused during film growth. After this H_2 cleaning, non-oxidized Cu is identified by Selected Area Electron Diffraction (SAED), which is shown in Supplementary Figure 1(b). Additionally, this annealing process also helps to create faceted holes on Cu films, which facilitate the ensuing observation of the oxide growth process from the cross-sectional view.

***In situ* oxidation.** Since the TEM only provides a 2D projection of its sample, discerning the initial oxidation process via observation from a top-down view of the film would be too difficult, given the thickness difference between the 60 nm thick Cu film and <10 nm thick Cu_2O island. Hence, an edge-on view of samples is needed for the direct observation of the oxide island growth process. The edge-on view is taken by first forming faceted holes with (100) and (110) side facets during the previously mentioned H_2 annealing process[1]. After creating these holes, samples were cooled down to 300 °C under H_2 until the thermal drift diminished to the point that HRTEM observation could be carried out. After the thermal drift attenuates, H_2 gas was cut off, then the gas line and the specimen chamber were pumped down to vacuum (~3×10^{-5} Pa). Afterward, O_2 gas was injected with a pressure of 0.3 Pa through another gas line, and *in situ* HRTEM observation was carried out on the (100) and (110) facets during the oxidation process. Real-time movies were recorded using a Gatan Orius 833 CCD camera with a frame rate of 5 frames/s. Supplementary Figure 1(c) shows a low magnification image of the sample after oxidation, with very small Cu_2O islands (marked by the arrows and circles) forming on the Cu film. The corresponding diffraction pattern shows that the oxide islands share cube-on-cube epitaxy with the Cu film.

3. Data process

Movie alignment. Although there are a lot of existing movie drift-correction methods, most of them are developed for purposes such as shaky video footage in camera videos recording daily life. These usually require unchanging markers, such as corners, to carry out image stabilization. However, for TEM movies, especially HRTEM movies, finding such unchanging markers is usually difficult. In this work, the Supplementary Movie tabilization is carried out using the Linear Stack Alignment with the SIFT plugin developed by Stephan Saalfeld in ImageJ, which uses the Scale Invariant Feature Transform (SIFT) algorithm[2]. To find markers for image stabilization or alignment, this algorithm compares continuously scanned frames for new features as the video is being processed. Hence, this algorithm is suitable for the alignment of HRTEM movies in which very small portions of images change from frame to frame.

Movie enhancement. After alignment, movies were cropped, rotated, and accelerated for easier observation using a combination of ImageJ and Python codes. To enhance image quality and increase Signal-to-Noise Ratios (SNR), movie frames were batch-enhanced by a home-made filtering code in ImageJ. This code used a combination of IFFT filtering and Wiener filtering to enhance the contrast of the image. Each frame of the movie was first Wiener filtered, then Fast Fourier Transformed (FFT) filtered, followed by adding masks to enhance Cu_2O diffraction spots and reducing noise signals in FFTs. This forms inverse FFTs (IFFTs) to get enhanced frames combining the filtered frames to form a new movie (shown in Supplementary Figure 12, **a-b**).

HRTEM image simulation. To confirm the experimentally observed HRTEM contrast, HRTEM image simulations were carried out using the DFT calculated structure models shown in Figures S8c and S9c. The multi-slice method was used to calculate the image, which was integrated into the software HREM. The image simulation parameters were chosen as follows: accelerating voltage = 300kV, spherical aberration coefficient of the objective lens (C3) = 0.01 mm.

4. DFT calculations

Computational results were achieved with Density Functional Theory (DFT) simulations, which were performed using the Vienna Ab Initio Simulation Package (VASP, version 5.4.4)[3, 4]. These simulations applied the Perdew-Burke-Ernzerhof (PBE) parameterization of the Generalized Gradient Approximation (GGA) functional[5]. Pseudopotentials used in these calculations were generated by the Projected Augmented Wave (PAW) method, with pseudopotentials for Cu and O labeled "Cu" and "O_s", respectively[6]. Preliminarily, multiple large sets of structures (Figures S17-S28) were screened to resolve the most favorable surface (γ), adsorption (E_{ads}), and diffusion (Δ) energies of each set. These sets – each of which was associated with a particular surface orientation, surface termination, and fraction of added layer formed – were initially evaluated in simulations featuring an energy cutoff (ENCUT) of 300 eV. Upon determining the most favorable structures for all sets and the diffusion mechanisms associated with them, subsequent simulations were performed to verify favorable structure energetics (performed for a subset of the above systems). These simulations featured an ENCUT of 400 eV, a dipole moment perpendicular to the exposed surface (IDIPOL = 3)[7, 8], and a Hubbard U of 7.5 eV on Cu 3d orbitals[9, 10, 11] modeled using the rotationally invariant Dudarev implementation of the Hubbard U model[12]. Over both sets of simulations, an electronic self-consistent cycle energetic tolerance of $1\text{x}10^{-5}$ eV, an ionic relaxation force tolerance of 0.02 eV/Å, and a 12x12x1 Monkhorst-Pack k-point grid (scaled relative to the number of Cu_2O unit cells in each surface dimension)[13] were used. The VASP Transition State Tools (VTST) software package was applied to perform Climbing Image Nudged Elastic Band (CI-NEB) calculations evaluating diffusion mechanisms[14, 15], applying 5 images per Minimum Energy Path (MEP).

Surfaces were constructed using the Cu_2O ($Pn\bar{3}m$, a = 4.25 Å) unit cell[16], applying either 3x3 or 3x4 supercell to exposed interfaces (xy-plane) and a vacuum of 20 Å to all structures (z-axis). Four layers of bulk Cu_2O underlie all surfaces, the bottom halves of which were constrained during relaxations. Oxide islands were grown on each surface incrementally as layers or adatoms, spanning an interfacial (xy-plane) area no larger than a single 1x1 Cu_2O unit. Oxide layer fractions, isolated in both interfacial directions, did not preserve Cu_2O stoichiometry. Thus, all surface energies were calculated via the following expression[7, 17]:

$$\gamma = \frac{1}{A_{surf}}\left(E_{test} - E_{ref} - \Delta N_{Cu}^{bulk} E_{Cu}^{bulk} - \frac{\Delta N_{O_2}}{2} E_{O_2}\right) \quad \text{(Supplementary Equation1)}$$

In Supplementary Equation 1, surface energies (γ) of simulated structures at shared temperature and pressure can be compared, given their surface areas parallel to the xy-plane (A_{surf}), reference Cu and O chemical potentials approximated with the DFT energies of the bulk FCC Cu unit cell (E_{Cu}^{bulk}) and the O_2 gas molecule (E_{O_2}), as well as the differences between the number of Cu (ΔN_{Cu}^{bulk}) and O_2 (ΔN_{O_2}) in tested and reference structures (normalized per unit cell or formula unit). DFT energies of tested (E_{test}) and reference (E_{ref}) surface structures are differently defined for particular γ calculations. For simulated Cu_2O surfaces with no island growth, E_{test} represents a studied flat surface and E_{ref} represents the bulk Cu_2O structure, thus γ calculates differences between surfaces and bulk Cu_2O. In contrast, simulated Cu_2O surfaces with grown oxide island layers compare the energetic differences between structures with (E_{test}) and without (E_{ref}, flat surfaces) those oxide layers, while per adatom structures are compared to bulk Cu_2O references.

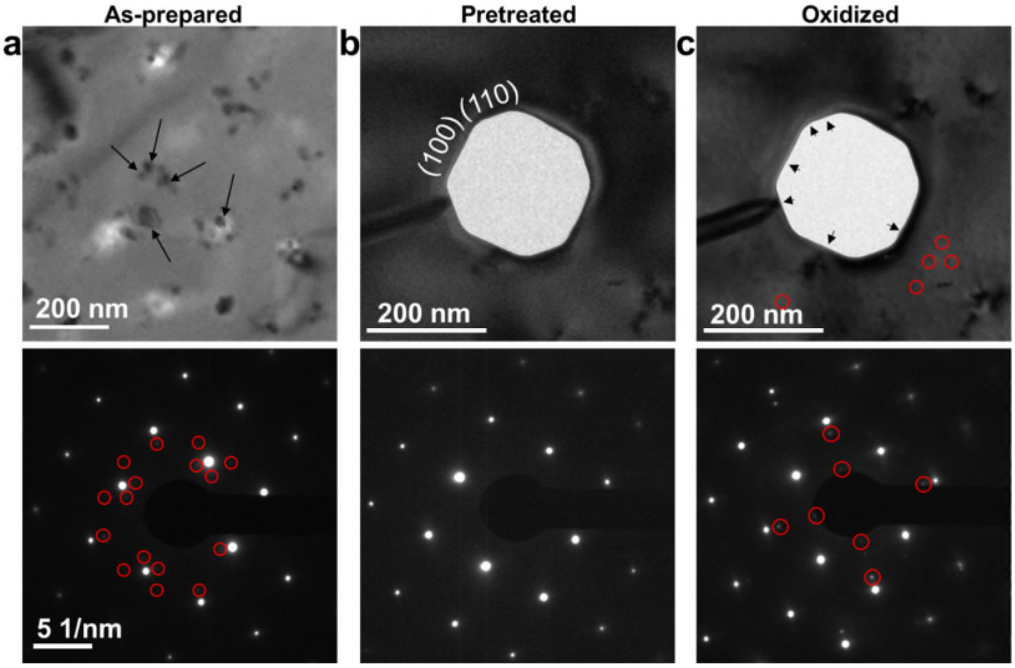

Supplementary Figure 1 | TEM images of the as-prepared, pretreated, and oxidized Cu film sample.

The upper row depicts Bright Field (BF) TEM images of a Cu film sample, while the lower row shows corresponding Selected Area Electron Diffraction (SAED) patterns. **a** The as-prepared Cu film sample has multiple native oxide islands (marked by arrows) confirmed by Cu_2O diffraction spots (circled) in the SAED pattern. **b** After pretreatment at 600 °C in 0.1 Pa H_2, the sample is reduced to a pure Cu film with faceted holes, while the clean SAED pattern reveals no oxides on the Cu film. **c** After oxidation at 300 °C under 0.03 Pa O_2, very small Cu_2O nano-islands formed (marked by arrows & circles); the SAED pattern shows a cube-on-cube epitaxy relationship with the substrate.

Supplementary Note 1: Additional experimental results for layer-by-layer Cu_2O growth along $Cu_2O(110)$

The geometry of the Cu_2O islands is determined by combining the top-down view, cross-section view images of the Cu_2O islands grown on Cu(100) and Cu(110) plane, shown in Supplementary Figure 2. Although $Cu_2O(111)$ plane has also been reported in previous papers as another common low-energy plane[18, 19, 20], the $Cu_2O(110)$ plane is more favorable over $Cu_2O(111)$ in oxygen-rich condition by calculations[21]. Previous chemical vapor deposition experiments have also suggested $Cu_2O(110)$ is the preferred growth plane.[22, 23, 24]

Cu_2O islands are found to grow layer-by-layer along $Cu_2O(110)$ on other substrate orientations. Supplementary Figure 3 and Supplementary Movie 2 shows multiple Cu_2O islands growing on a Cu(100) film flat surface (terrace) and a Cu(110) facet. At the upper left corner of each image of Supplementary Figure 3, a Cu_2O island grows epitaxially on the Cu(110) facet, namely along the $Cu_2O(110)$ plane to form a rectangular shape. At the bottom of each image of Supplementary Figure 3, three Cu_2O islands were observed to grow on the Cu(100) flat surface vertical to the electron beam direction, as can be identified by their Moiré fringes. These islands have a square shape with $Cu_2O(110)$ facets. As the islands grow, their Moiré fringe boundaries expand layer-by-layer, maintaining the outer rim of $Cu_2O(110)$ facets. Note that the exteriors of the islands (closer to the faceted hole) grow quicker than their interiors, indicating more preferred oxide growth around the faceted holes. At the center-right of each image of Supplementary Figure 3, two Cu_2O islands grown on a Cu(100) facet were observed. Although only $Cu_2O(100)$ lattice fringes were observable due to their orientation relative to the electron beam, island side facets have small surface steps that proceed along $Cu_2O(110)$ planes. Supplementary Figure 4 and Supplementary Movie 4 show another example of a Cu_2O island growing on the Cu(100) terrace and then expanding into the vacuum. As shown by the Moiré fringes and the expanding part of the island, the island preferentially grows along $Cu_2O(110)$ planes. Supplementary Figure 5 and Supplementary Movie 5 show that in the later oxidation stage, when the oxide island is large, the oxide is still growing layer-by-layer along $Cu_2O(110)$. These results indicate layer-by-layer growth along $Cu_2O(110)$, similar to that shown in Figure 1, is the preferred oxide growth process.

Supplemenarty Figures 6-8 show additional details of Supplementary Movie 1, validating the diffusion-limited oxide growth process. Supplementary Figure 9 shows proof of missing row reconstructed surfaces of Cu(100) during Cu_2O growth in Supplementary Movie 5. Supplementary Figure 10 shows additional results involving the Cu||Cu_2O interface due to interface diffusion.

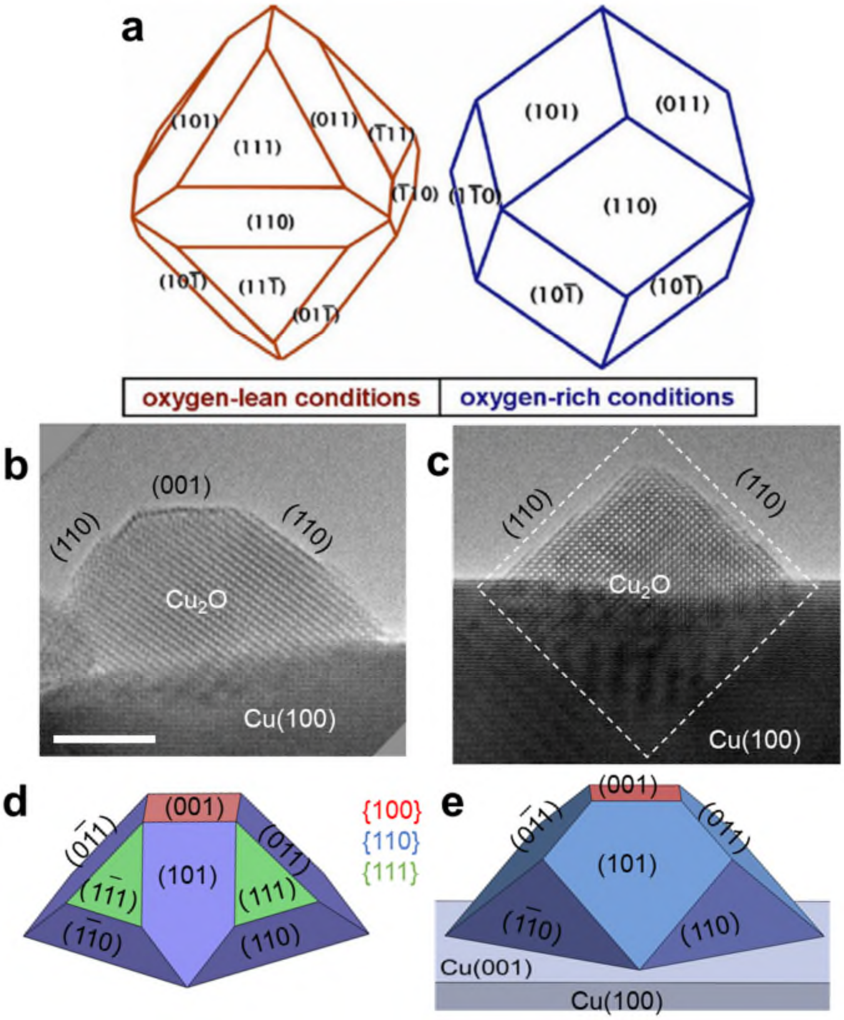

Supplementary Figure 2 | 3D morphology of the Cu_2O islands.

a Wulff construction of the Cu_2O crystal under oxygen-lean (left) and oxygen-rich (right) conditions taken from past literature[21]. **b** HRTEM image of Cu_2O island grown on Cu(001) facet that is parallel to the e-beam, providing an edge-on view of the island. **c** HRTEM image of Cu_2O island grown on Cu(100) surface that is perpendicular to the e-beam, providing a top-down view of the island. Scale bar: 2 nm. **d-e** Possible 3D morphology of the Cu_2O island under oxygen-lean **(d)** or oxygen-rich **(e)** conditions. The Cu_2O{100}, {110}, and {111} surfaces are colored red, blue, and green, respectively. In both conditions, the experimental observed new layers are projections of grown new layers on Cu_2O(011) surfaces.

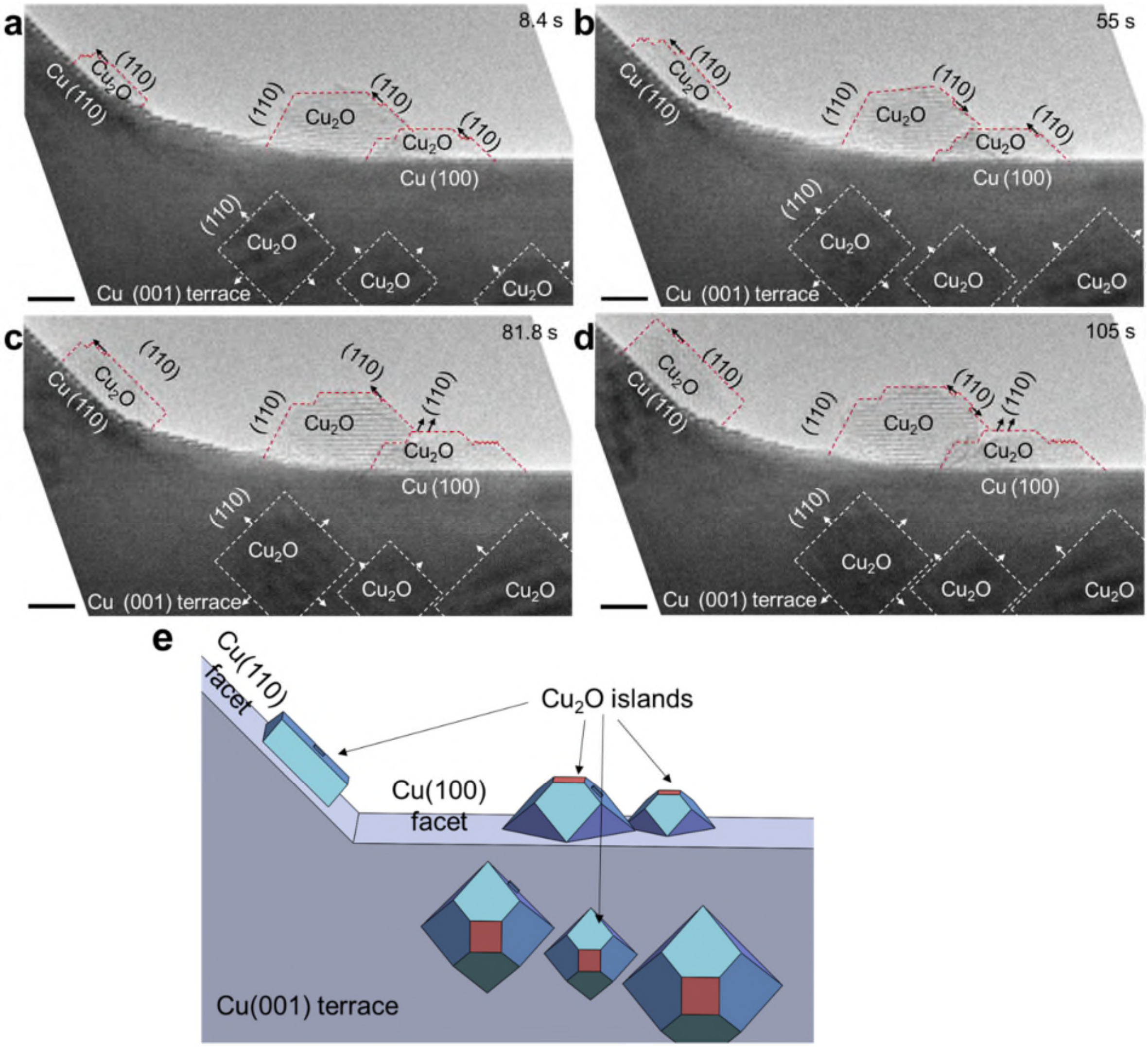

Supplementary Figure 3 | Layer-by-layer growth of Cu_2O on Cu(100) and (110) surfaces. **a-d** Snapshots of Supplementary Movie 2 showing layer-by-layer growth of Cu_2O along the Cu_2O(110) plane over all of the observed Cu facets at 300 °C and under Po_2 = 0.3 Pa. One rectangular shaped Cu_2O island is observed to grow along the Cu_2O(110) plane of the Cu(110) facet on the upper-left portions of the images. Two Cu_2O islands were growing on the Cu(100) facet along the Cu_2O(110) plane, located on the center-right portions of the images. Three Cu_2O islands were growing on Cu(001) film terraces and were identified by Moiré fringes caused by overlapping Cu_2O and substrate Cu(001) lattices. The shape of the Moiré fringe zone shows Cu_2O(110) orientations. Scale bar: 2 nm. **e** The corresponding model showing the 3D geometry of the sample. The Cu_2O{100}and {110} facets are colored red and blue, respectively.

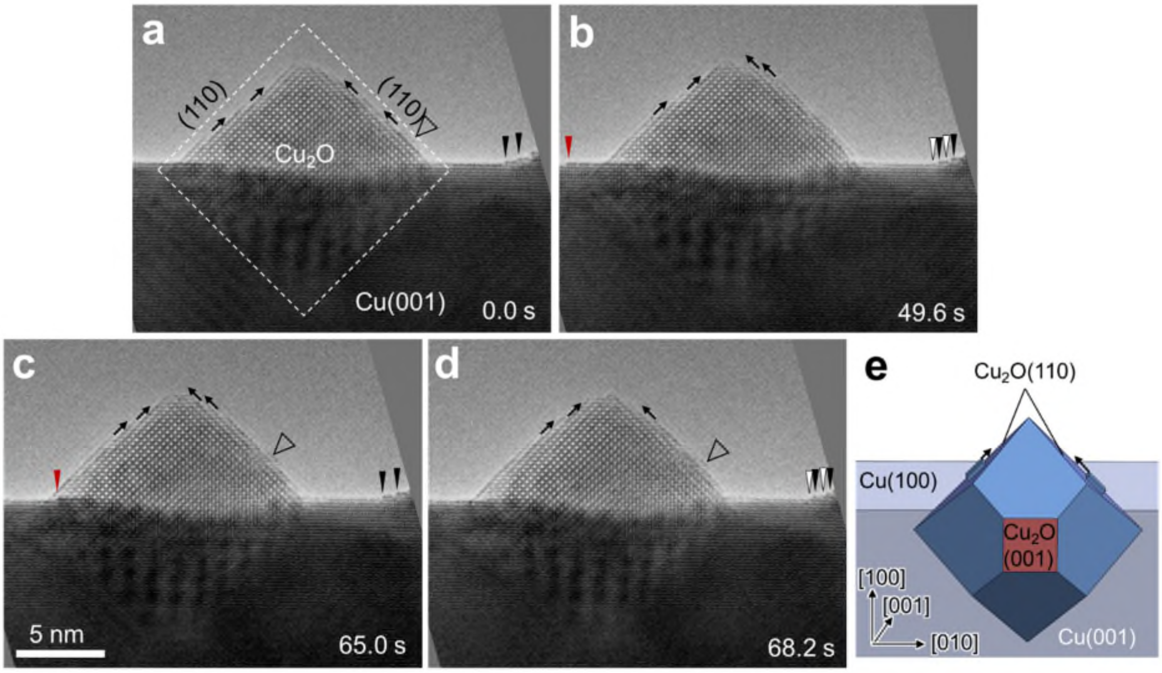

Supplementary Figure 4 | Cu_2O island growth on Cu(001) film surface

a-d Snapshots from Supplementary Movie 3 showing a Cu_2O island growing on the surface of Cu(001) film perpendicular to the *e*-beam, extending into the vacuum at 300 °C and under P_{O_2} = 0.1 Pa. The suspending part shows the adatom growth of Cu_2O along the Cu_2O(110) plane (marked by the arrows). **e** Corresponding 3D model showing the geometry of the Cu_2O island on the Cu film. Cu_2O {110} and {100} surfaces are colored blue and red, respectively. Note the Cu_2O island grows much slower than the one in Supplementary Movie 1 due to decreased oxygen partial pressure. The hollow triangles marked the nucleation of a new layer. Note the retreat of the surface steps on the Cu(100) facet marked by narrow triangles. Black and red triangles correspond to the current positions of the step edges, while white triangles correspond to their previous positions. Moiré fringes are caused by the overlapping of the Cu_2O island and the Cu film. Scale bar: 5 nm.

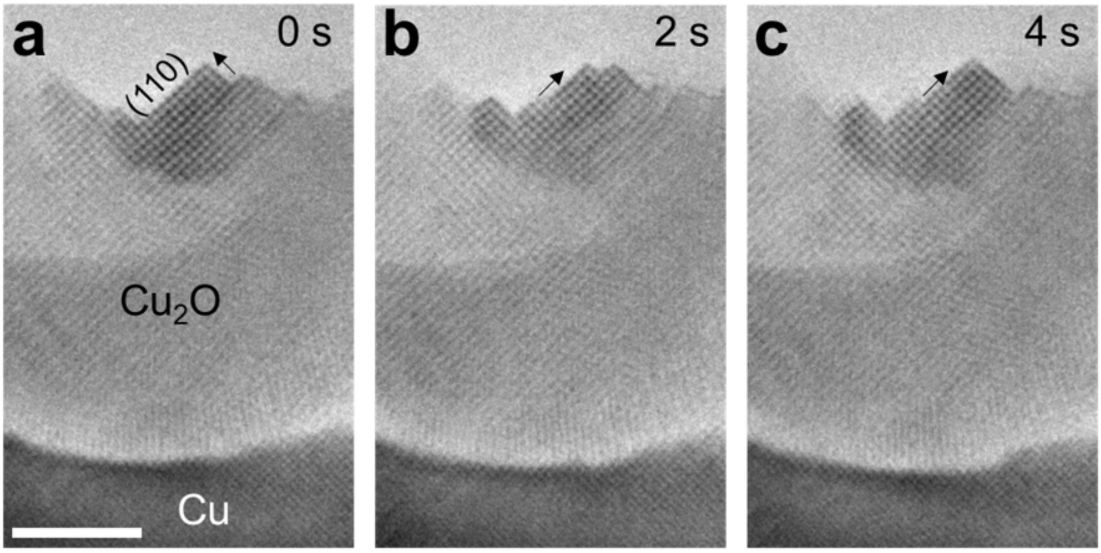

Supplementary Figure 5 | Layer-by-layer growth of Cu_2O in later oxidation stage.
Snapshots of Supplementary Movie 4 showing layer-by-layer growth of Cu_2O along $Cu_2O(110)$, even when the oxide is large at 300 °C and under Po_2 = 0.3 Pa. Scale bar: 5 nm.

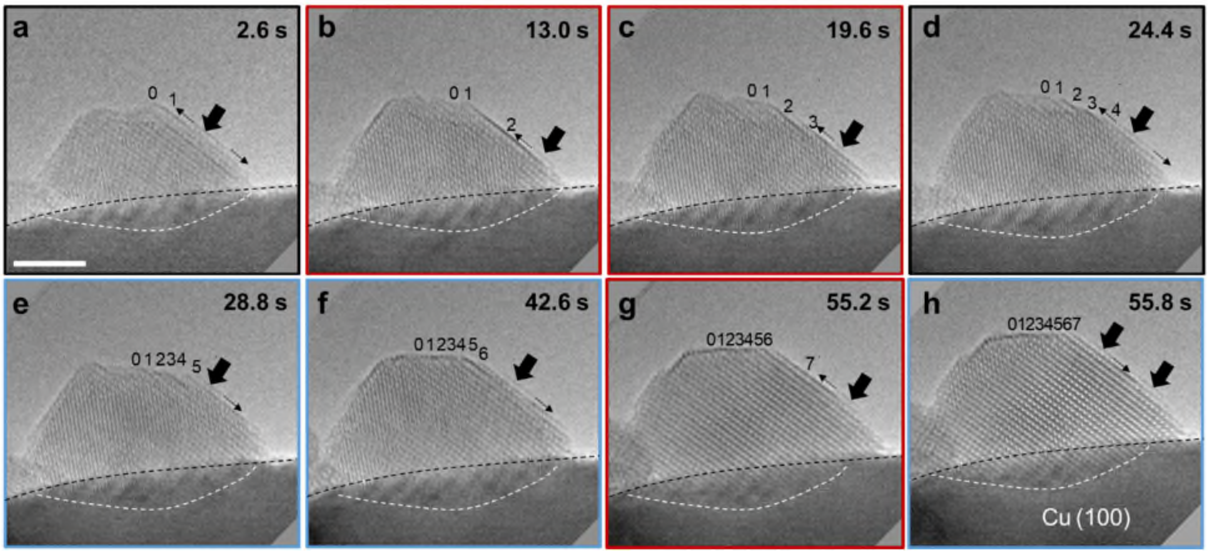

Supplementary Figure 6 | Random nucleation sites of each new Cu_2O monolayer.
a-**f** Snapshots of frames when the new layer is first visible for the first 6 new layers. 2 layers are nucleated at the center (**a** & **d**, black borders), 2 layers are nucleated at the bottom (**b** & **c**, red borders), and 2 layers are nucleated at the top (**e** & **f**, blue borders). The large arrows show the nucleation positions of the layers, and the small arrows mark the main growth directions of the layers. **g-h** Snapshots of nucleation sites of the 7th layer, indicating the new layer first nucleates near the bottom **(g)**, and then another nucleation event involving the same layer occurs near the top **(h)**. This is possibly caused by the relatively larger size of the substrate layer (6th layer). Scale bar: 2 nm.

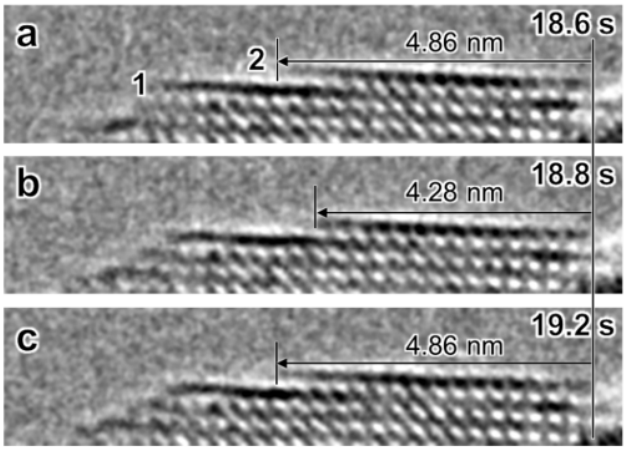

Supplementary Figure 7 | Oscillatory growth of Cu_2O(110) monolayer.

Back-and-forth oscillatory growth is observed in the growth front of the new layer (layer 2). The images are taken from the boxed area in Figure 1f and are contrast-enhanced.

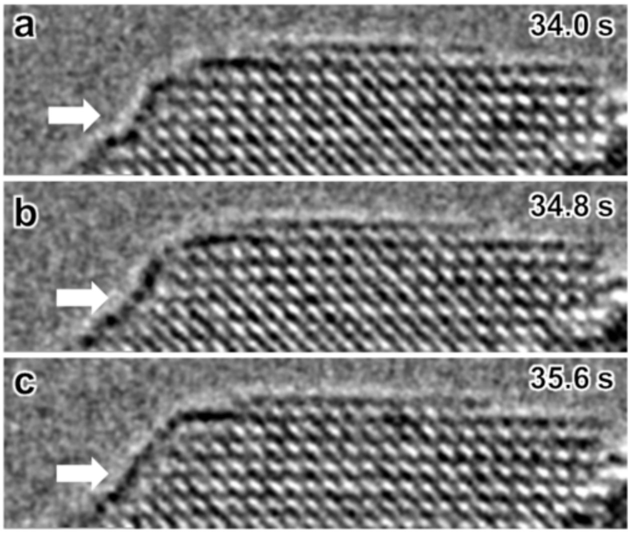

Supplementary Figure 8 | Adjustment of the top of the island during oxidation.

The images are cropped from the boxed area in Figure 1d and are contrast-enhanced. The arrowed area in **(a)** was a Cu_2O(110) stepped surface, and gradually adjusted to a flat Cu_2O(100) surface in **(c)**.

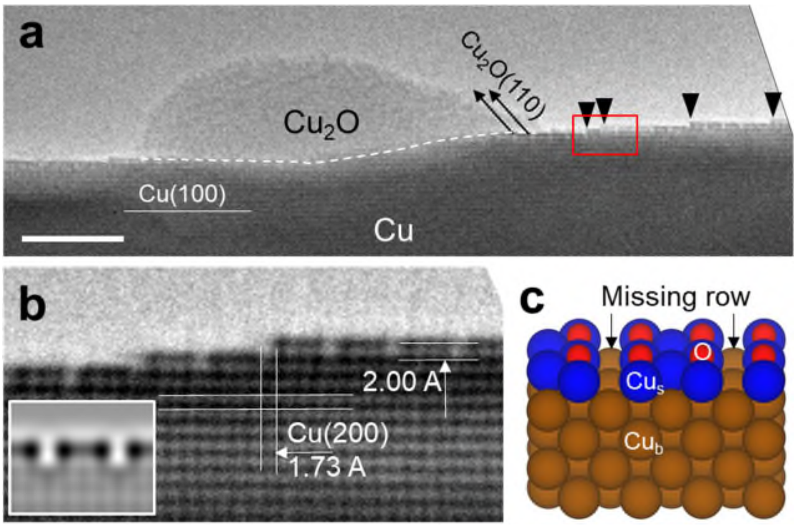

Supplementary Figure 9 | Reconstructed Cu(100) surface during oxidation.

a HRTEM image of Cu_2O island growth shown in Figure 3a. Scale bar: 5 nm. **b** Enlarged HRTEM image of the boxed area in **(a)** shows that Cu(100) is reconstructed during oxidation, while the inset shows the simulated HRTEM image of the Missing Row Reconstruction (MRR) surface. **c** Corresponding atomic structure of the MRR on the Cu(100) surface for HRTEM image simulation, tilted slightly upward from the direction depicted by the electron beam to give a 3D perspective of the reconstructed feature.

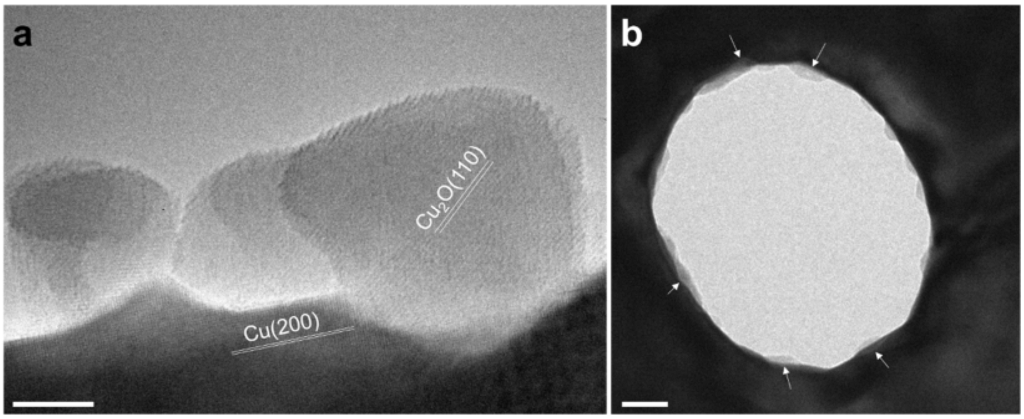

Supplementary Figure 10 | Cu_2O islands formed via interface Cu diffusion.

a HRTEM image of less distinctly faceted Cu_2O islands with a concave Cu/Cu_2O interface, indicating the outward diffusion of substrate Cu leading to the growth of Cu_2O. **b** Lower magnification BF-TEM image showing the shape of the entire hole after oxidation. Hole edges change from straight, well-defined facets (Supplementary Figure 1) to concave Cu/Cu_2O interfaces formed from grown Cu_2O islands, as is marked by arrows. Scale bars: **(a)** 5 nm, **(b)** 20 nm.

Supplementary Note 2: E-beam effect

E-beam effect is a general issue for *in-situ* TEM studies. The e-beam effect includes knock-on damage and radiolysis. Knock-on damage is caused by the displacement of atoms in the sample by momentum transfer with the electrons. Radiolysis is caused by electrons modifying the chemical bonds in the sample. Besides, for E-TEM, ionization of gas molecules caused by the interaction between free electrons and the gas is another major e-beam effect.[25, 26] We account for these effects as follows:

Knock-on damage – For Cu, the threshold value of incident energy for displacement is 420 keV[25], which is above our experimental condition (300 keV). Hence, knock-on damage can be omitted.

Radiolysis – For oxide such as Cu_2O, a strong electron beam has been reported to be able to cause the reduction of the oxide[25, 27]. To avoid this effect, a series of e-beam dosage has been tested in our experiment, the e-beam dosage we chose is as follows: acceleration voltage: 300 keV, Emission current: 3 μA, e-beam dosage (measured without sample): ~138 nA/um^2 or 8.6 × 10^5 $e/nm^2 \bullet s$, this is lower than the dosage normally used for HRTEM imaging from literature reports (~1-5 × 10^5 $e/nm^2 \cdot s$)[28]. Under this e-beam dosage, no obvious change in the size of the Cu_2O islands was observed in vacuum over 3~5 minutes of e-beam exposure, while the Cu_2O islands grow quickly when O_2 was injected. This further indicates that the observed oxide growth is not significantly affected by the e-beam. Since low e-beam dosage decreases the signal-to-noise ratio of the images, the Supporting Movies and images are averaged every two frames after alignment and signal enhanced.

Ionization of gas molecules - the ionization of gas molecules would help dissociate O_2 molecule to O atoms, which could increase the reactivity of the oxidation experiment. Some previous experiments[27, 29] reported oxide growth of Mg in vacuum at room temperature due to the electron beam assisted ionization of residual O_2 inside the TEM chamber. According to the oxide phase diagram with temperature and oxygen partial pressure, the threshold oxygen pressure for oxide formation increases with increasing temperature[17]. Since the aforementioned experiments were carried out at room temperature, the vacuum level of the TEM chamber is much higher than the threshold partial pressure. Hence, *e*-beam induced oxide growth could be observed under vacuum. In comparison, our oxidation experiment is carried out at 300 °C in this work, under which condition the oxygen partial pressure needed for oxide formation is much higher. In this work, to remove any remaining O_2 or contamination in the ETEM, the ETEM chamber was baked before the experiment, the sample holder is plasma cleaned, tools in contact with the sample are UHV-cleaned, and the pretreated sample with clean Cu surfaces are kept in H_2 before the oxidation experiment. As a result, no oxide growth or even surface reconstruction is observed on the sample under vacuum under the same e-beam dosage (Supplementary Figure 11). In comparison, surface reconstruction is observed under 0.07 Pa O_2 and above, and Cu_2O nucleation and growth is observed at ~0.3 Pa, indicating the observed oxide growth is mainly caused by the injected O_2.

Our earlier experiments have compared the Cu_2O island size and density evolution during Cu oxidation with and without electron beam[30], the result shows that although the electron-beam could increase the reaction rate by approximately a factor of four, the oxide island shape and distribution with and without e-beam remains consistent, indicating the e-beam effect have limited effect on the oxide growth mechanism.[30]

After *in situ* HRTEM observation, oxide islands were observed on both *e*-beam illuminated and un-illuminated areas with similar geometry and orientations. For example, the islands shown in Supplementary Movie 1 and S3 are moved from areas that were not illuminated by the *e*-beam, but still shows clear facets of $Cu_2O(110)$. All these results indicate that, even though the electron beam might alter the reaction rate, the *e*-beam should not have significantly affected the observed oxide growth mechanism.

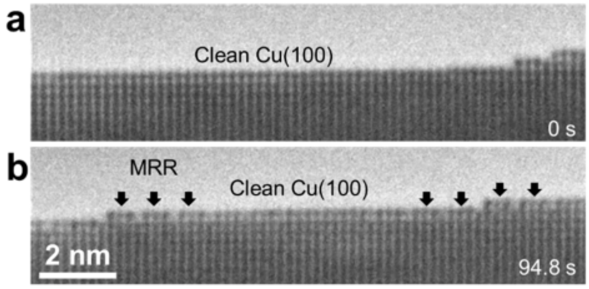

Supplementary Figure 11 | Formation of surface reconstruction on Cu when O_2 was injected.

Time sequence showing the formation of missing row reconstruction on Cu(100) surface since the gas change from vacuum to 0.1 mTorr O_2. The gradual formation of surface reconstruction under O_2 indicates the residual oxygen in the ETEM chamber under vacuum is below the threshold oxygen level for oxide formation even with the assistance of the *e*-beam.

Supplementary Note 3: Data analysis

1. Measuring growth trajectories from *in situ* HRTEM movies

To quantitatively measure the atomic-scale growth trajectory of each layer, kymographs of each layer from the filtered movie were extracted. Kymographs are a way to represent a dynamic process of a movie on a single image. They can be seen as position-time (x-t) scans of Supplementary Figure 12b, where the intensity along a given line is plotted for all images of a stack. Each time point gives an intensity line, plotted along the x-axis of the kymograph. These lines are stacked along the y-axis for all frames. Thus, in the kymograph, the evolution of the intensity (x-axis) of a selected line over time (y-axis) or the duration of a movie is shown in one static image. In this work, kymograph measurements of each of the studied 7 layers are extracted using an ImageJ macro, which collinearly renders the scanned lines of each layer sharing the same x positions and times. This makes the kymograph measurements of each layer comparable. To enhance the SNRs of kymograph measurements, line-scans of each selected line are summed together at widths of 5 (rather than 1) pixels (1 Å). The acquired kymographs of each layer are shown in Supplementary Figure 12(c-i).

After getting the kymographs, boundaries of the layers in the kymographs detected by contrast differences are plotted in corresponding colors in Figures S10(c-i). As shown in Supplementary Figure 12(c), the stripes in the kymograph measurements came from the lattice contrast in the HRTEM images and they are quite straight, indicating that the frames are well aligned. The left and right coordinates of the kymograph measurements are then plotted together in Supplementary Figure 10(j); the length evolution of each curve, defined by the distance between the left and right coordinates, is plotted in Supplementary Figure 12(k).

Using this approach, the size evolution of the overall height and width of the Cu_2O island, as well as the growth rate along <110> direction, are also measured and plotted in Supplementary Figure 13.

3. Growth rate analysis

After getting the quantitative growth data from each monolayer plotted in Supplementary Figure 12(k), an in-depth analysis of these numerical data is performed using mathematical methods.

To analyze the growth rate of each layer, the projection lengths of each layer are plotted using the relative time from the time when each layer is nucleated in Supplementary Figure 13(d). This plot clearly shows that each curve shares a similar overall trend. To get this trend, data fitting is performed using power-law fitting $l^n = At$. The fitted n of each layer is close to 3. After this, cubic fitting $l^3 = At$ is performed on each layer. The fitting results plotted in Supplementary Figure 14 show quite consistent fitted A values, except for the 7th layer. This exception is because the 7th layer has 2 nucleation sites shown in Supplementary Figure 12(g-h), which leads to a faster growth rate. By plotting the cubed length l^3 versus time in Supplementary Figure 13(e), all curves are very close to straight lines with similar slopes, further substantiating the fitting result.

4. Statistical analysis

Besides similar overall trends, Supplementary Figure 13(d) also showed several simultaneous changes among several layers. To validate these concurrent changes, break-point analysis using a multivariate (multiple simultaneously assessed dependent variables or *l*) time-series statistical model was performed. Breaks in measured cubed lengths of different oxide monolayer (l_i) ranges, namely from source (*Se*) to sink (*Sk*), over *t* are measured with corresponding growth rates $(dl^3/dt)_j$, shared breakpoint dummy variables ($\delta_{t=break}$), and residual errors ($\varepsilon_{i,t}$) using the multivariate regression formalism in Supplementary Equation 2. Further information on the statistical tests employed, as well as their explicit connections to experimental observations, are described in Supplementary Note 4.

$$\sum_{i=Se}^{Sk} l_{i,t}^3 = \sum_{j=Se}^{Sk} \left(\frac{dl^3}{dt}\right)_j t + l_{i,t=0}^3 + \delta_{t=break} + \varepsilon_{i,t} \quad \text{(Supplementary Equation 2)}$$

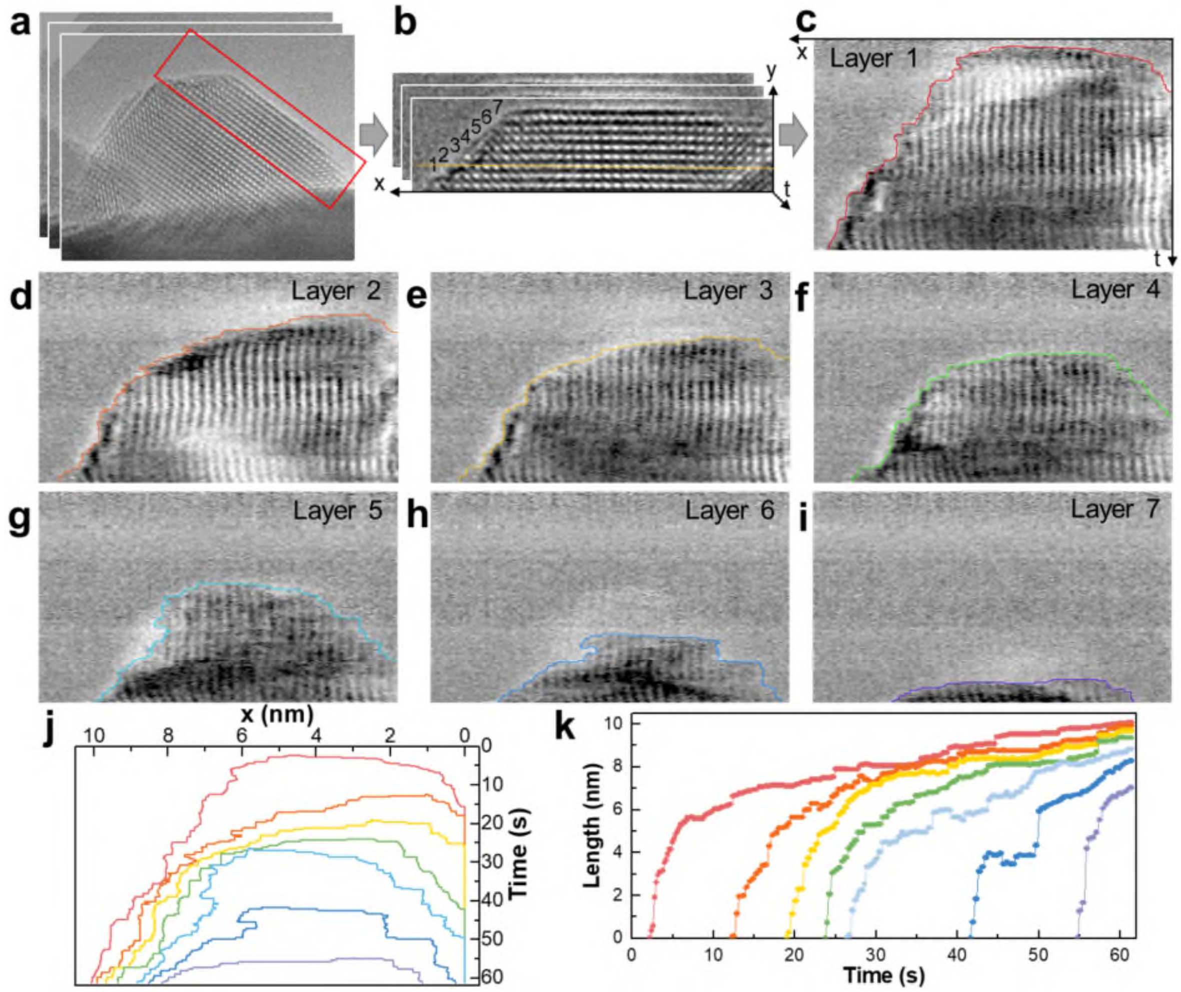

Supplementary Figure 12 | Extracted growth profile of each layer.

a Snapshot of the movie. **b** Enhanced image of the boxed area in **(a)**; layers 1-7 are labeled on the left. **c-i** Extracted kymographs of layers 1-7, respectively, with colored curve outlines showing the growing island shapes detected from the kymograph. **j** Plots of the growth

trajectories of each layer based on the detected outlines. **k** Plots of projection length of each layer with time calculated from **(j)**.

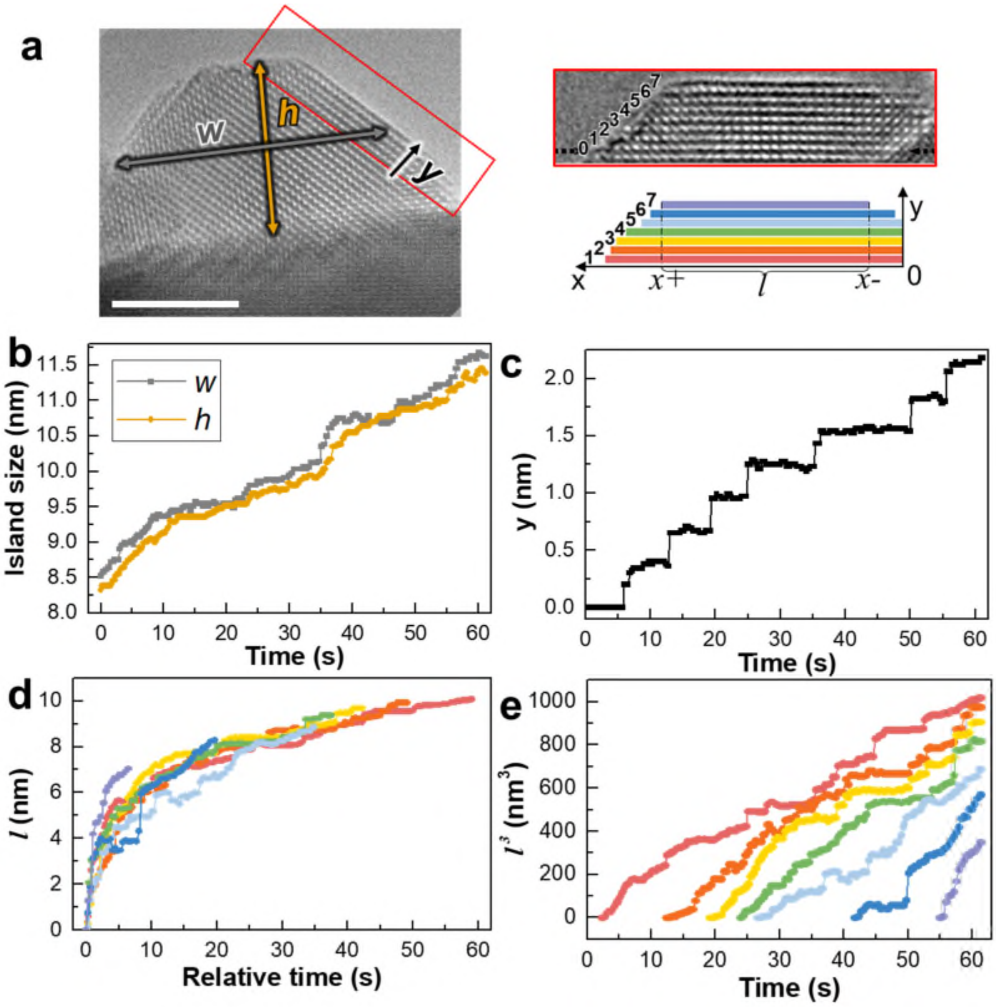

Supplementary Figure 13 | Additional growth rate analysis results.

a Schematic showing the definition of the measured data. **b** A plot of the overall island size versus time. The width of the island (*w*, colored gray) is measured at the position shown in **(a)** to avoid influence from the adjacent island on the lower-left corner. The height of the island (*h*, colored yellow) is measured from the center of the island. **c** A plot of the vertical growth rate of the new layers vertical to $Cu_2O(110)$. **d** A plot of the projection length (*l*) of each layer versus the relative time since each layer nucleates. The overall trend shared by these layers is very similar. **e** A plot of l^3 versus time; all curves are close to straight lines, further substantiating the cubic fitting result.

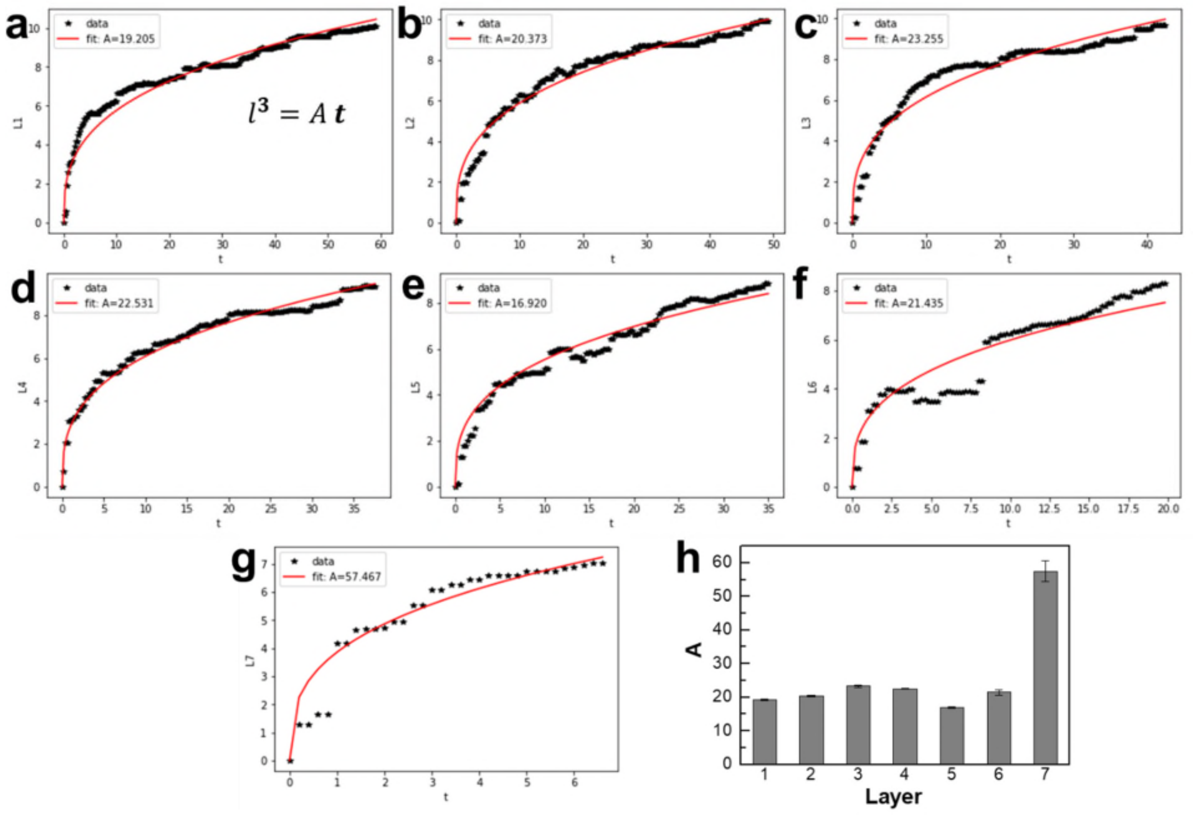

Supplementary Figure 14 | Cubic fitting result of each layer using function l^3=*At.*

a-g Fitted curves (red) depicting the growth of layers 1-7, respectively. The vertical axis of each plot is projected length (*l*, nm), while the horizontal axis is time relative to the nucleation of each layer (*t*, s). **h** The plot of the fitted *A* values. The *A* values are very consistent throughout the first 6 layers, while the 7th layer *A* value is significantly larger in conjunction with the observation of two nucleation sites on that layer. The standard error of the fitted A are plotted in the figure.

Supplementary Note 4: Statistical analysis of the growth rate

Cubed lengths (l^3)[31, 32, 33] of each growing oxide layer shown in Supplementary Figure 13 are compared over time (t) via time series analysis to evaluate when breaks in otherwise continuous layer growth occur. Statistically defined structural breaks[34] identified in this analysis (Supplementary Figure 15) are equated to three types of experimental observations and associated reaction processes – intralayer effects, interlayer nucleation effects, and concerted diffusion events. Intralayer effects (single-layer) describe oxide nucleation impact on the growth rate of the current top oxide layer (T1-T2 in Supplementary Table 1). Interlayer nucleation effects are interactions over multiple formerly grown oxide layers coinciding with the nucleation of a new oxide layer (N2-N7 in Supplementary Figure 15). Concerted diffusion events supply formed bottom layers of oxide islands with Cu sourced from island top layers (P1-P6 in Supplementary Figure 15).

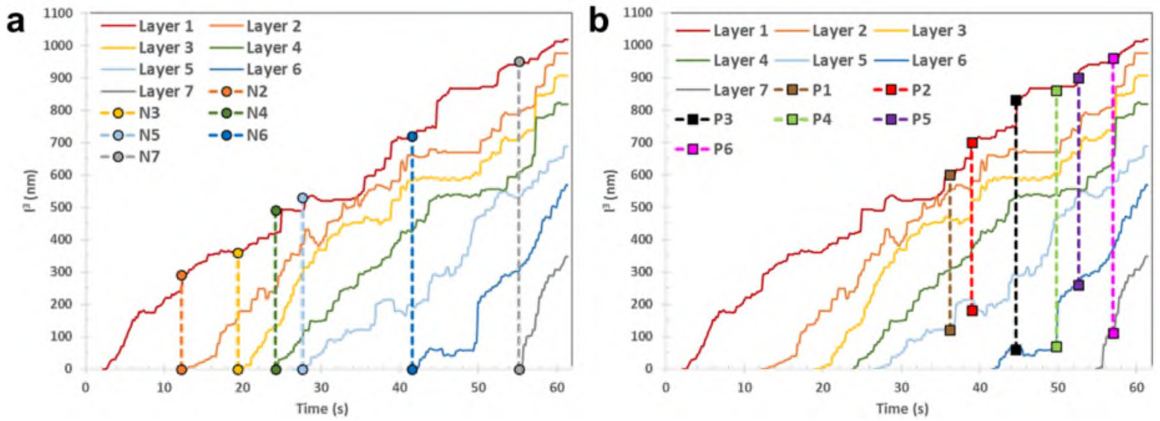

Supplementary Figure 15 | Initial times and oxide layer range guesses over the tested structural breaks.

Tested breaks can be linked to **(a)** interlayer nucleation events (N points) or **(b)** concerted diffusion events changing sourced Cu position (P points). Dotted lines span layers through which breaks are proposed to occur at tested times. Breaks are respectively labeled as N2-N7 (connected circles) or P1-P6 (connected squares), while initial breakpoint times (t) corresponding to these plots are listed in Supplementary Table 1. Note that Layer 1 begins growth at $t = 2.2$ s and serves as a reference for the other breakpoints. Overall initial guesses for P1-P6 are estimated as averages of apparent breaks on single layers, which are treated as breakpoints sharing a single time value in initial estimates.

Univariate statistical models (one l vs. t relationship) can detect how reactions affect single oxide layers, inferring oxide growth breaks given initial guesses for breakpoint times. The supremum-Wald F test (sup-Wald or sup-F test) accomplishes this by iteratively performing Chow breakpoint tests over an estimated window of time in which a breakpoint is expected. This yields a local maximum F test statistic value within the window that infers the breakpoint[35, 36]. All breakpoints on single layers are sup-F tested via a screening procedure (Supplementary

Figure 16 and Supplementary Table 1), finding oxide growth breaks within several criteria governing *F* statistic magnitude and other data characteristics.

Supplementary Table 1 | Breakpoint guesses for intralayer oxide nucleation to growth transitions (T), interlayer nucleation (N) of new oxide layers, and concerted diffusion through layers or change in Cu position (P).

Transition		Nucleation		Position	
Name	Break (s)	Name	Break (s)	Name	Break (s)
T1	6.8	N2	12.2	P1	36.2
T2	16.6	N3	19.4	P2	39
		N4	24.2	P3	44.6
		N5	27.6	P4	49.8
		N6	41.6	P5	52.6
		N7	55.2	P6	57.2

Thin-film growth models feature competing diffusion mechanisms explaining mass transfer between or across surface and subsurface film layers. Reaction conditions or nucleation density can trigger sudden switches in the relative reaction rates of competing mechanisms, causing apparent breaks or oscillations in film growth at switching thresholds.[32, 37] Figure 1 depicts layer-by-layer Cu_2O growth with stepwise oscillations over the growth fronts of each layer, especially in the total projection lengths of each oxide layer with time (Figure 1f). Relative to experimental observation, these oscillations are evaluated with structural break tests on univariate statistical models.[34] Given that Cu supply is rate-limiting in these reactions, such oscillations are linked to Cu transport to growth fronts via diffusion from sources. For interlayer effects, Cu generally diffuses from formerly grown (lower) island layer sources to nucleating (top) oxide layers (N2-N7, labeled by nucleating island). During concerted diffusion events, Cu generally diffuses from top (5-7) oxide layer sources to bottom (1-2) or middle (3-4) layers (P1-P6, labeled by sequenced event). Intralayer events are linked to transitions (T) between the surface reconstruction and subsequent growth stages of single oxide layers, sourcing Cu from grown oxides on layers to currently growing layer components. For layers 1 (T1) and 2 (T2), these transitions occur over sufficiently long periods and are large enough to be tested. All statistically validated breakpoints are shown in Supplementary Figure 16, which indicates that single concerted diffusion and interlayer nucleation events do occur at slightly different times over multiple layers. Regardless of whether this occurs due to measurement error or reaction process characteristics, direct cross-sectional comparisons of single breakpoints over multiple layers is not always possible and alternative statistical techniques must be used.

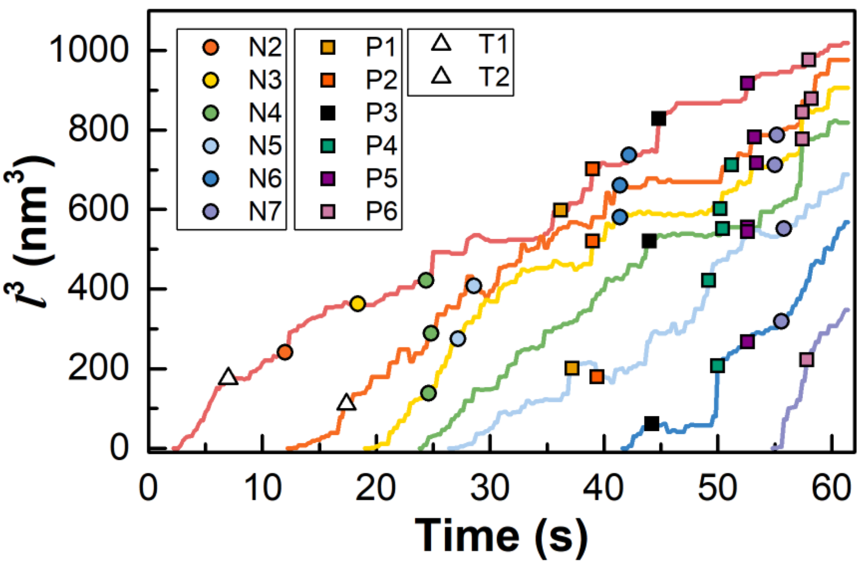

Supplementary Figure 16 | Statistically verified structural breakpoints.
Statistically verified structural breakpoints corresponding to new oxide layer nucleation effects (N) on former layers (circles), Cu position changes (P) from interlayer diffusion (squares), and transitions from oxide nucleation to growth (T) stages of single oxide layers (hollow triangles). Color coding implemented in past figures for enumerated oxide layers and breaks is preserved here.

The Chow test can determine whether a single known breakpoint between two contiguous fitted linear relationships occurs at a proposed time shared by them. Linear relationships are fitted from data taken before (*a*, sample size N_a) and after (*b*, sample size N_b) a tested breakpoint, and are compared with a single linear relationship encompassing both relationships ($a+b$). Comparisons consider the total number of fitted coefficients (*k*) of linear relationships containing data taken before and after the breakpoint. Island length (*l*) growing in two dimensions scales with time (*t*) as $l \sim t^{1/3}$,[32, 37] while structural breaks in trends over time are usually designed to handle trends linear in their independent variables.[34] Therefore, all data (from Figure 1f) is scaled to an $l^3 \sim t$ relationship in these tests, and initial cubed length ($l_{t=0}^3$) and growth rates (dl^3/dt) are fitted to yield their summed squared residual (SSR) measurements. A linear model for regression (error ε), a hypothesis test (null and alternate hypotheses H_0 and H_A, respectively), and an *F* test statistic are used to evaluate null hypothesis rejection in the Chow test:

$$l^3 = \left(\frac{dl^3}{dt}\right) t + l_{t=0}^3 + \varepsilon \;\; | \;\; F = \frac{[SSR_{b+a} - (SSR_b + SSR_a)]/k}{(SSR_b + SSR_a)/(N_b + N_a - 2k)} \quad \text{(Supplementary Equation 3)}$$

$$H_0 \colon l_{t=0,b}^3 = l_{t=0,a}^3 \; AND \; (dl^3/dt)_b = (dl^3/dt)_a$$

$$H_A \colon l_{t=0,b}^3 \neq l_{t=0,a}^3 \; AND/OR \; (dl^3/dt)_b \neq (dl^3/dt)_a$$

However, the Chow test cannot directly assess structural breaks in this data, given previously mentioned variations in breakpoint time. Thus, approximate ranges of initial guesses for structural breakpoints are estimated. Chow *F* tests can be completed over domains (or windows, from i_1 to i_2) of data in single tests to evaluate breakpoints over estimation windows,

determining whether – at any point in that window – a breakpoint can be inferred by rejecting H_0. Therefore, the supremum (*sup*; equivalent to a maximum in this study) F test statistic value within a window will infer structural breaks. This test evaluates the statistical significance of adding a linear coefficient to fit data, thus it is considered a Wald (sup-Wald) test. Its null and alternate hypotheses, as well as its test statistic (sup-F), are listed below relative to a statistical significance threshold (L):[35, 36]

$$H_0: \sup F < L \ \ | \ \ H_A: \sup F \geq L \ \ | \ \ \sup F = \sup_{i_1 \leq i < i_2} F_i \qquad \text{(Supplementary Equation 4)}$$

Data window sizes for evaluating structural breaks are partially selected by considering practical limitations of available data, namely that studied l^3 vs. t data are incrementally spaced (Δt = 0.2 s) and initial estimates for breakpoints can be as close as 1.8 s (N7-P6, 10 data points) from one another. Additionally, data window selection is statistically justified by the determination of a local maximum of each concave F-statistic vs. t relationship, or probabilistic sample space formed by completing Chow tests with incrementally changing breakpoints over windows. This incremental testing constitutes the sup-Wald F method, which is performed over each pair of data segments immediately preceding and succeeding each initial breakpoint estimate. Application of sup-Wald F tests over all proposed breakpoints develops a screening procedure for breakpoints, the results of which are visualized in Supplementary Figure 14 and summarized in Supplementary Table 2. These sup-Wald F tests reject H_0, which states that there is no break in slope or intercept of a univariate dependent variable (l^3) fitted to a linear model over t via ordinary least squares regression. Over single screened layers, the end of the segment succeeding (EP_i) each initial breakpoint guess (BP_i) was the subsequent breakpoint (BP_{i+1}) to be tested ($EP_i = BP_{i+1}$), while the beginning of each subsequent segment (SP_{i+1}) was the initial guess for each starting breakpoint (BP_i) ($SP_{i+1} = BP_i$). Variations of fitted breakpoints from initial guesses and failure to reject H_0 can change initialized equivalences.

Supplementary Table 2 | Numerical summary of univariate screening structural break analysis.

For each layer (1-7) and each denoted break, start (S_P) and end (E_P) points of each fitted segment tested for structural breaks, fitted breaks (B_P) resulting from testing, numbers of samples before (N_{sb}) and after (N_{be}) each fitted break, F statistic (F) and p-values (p) of each test, coefficients of the fitted sample before [R^2_{sb}] and after [R^2_{be}] each sample, and slopes (k) of each fitted sample.

Layer 1											
	SP (s)	EP (s)	N_{sb}	N_{be}	BP (s)	F	p	R^2_{sb}	R^2_{be}	k_{sb}	k_{be}
T1	2.2	12.2	24	27	7	305.62	2.200E-16	0.9754	0.92	41.059	15.36
N2	7	19	25	36	12	216.54	2.200E-16	0.92	0.8923	15.3599	11.785
N3	12.4	24.8	30	33	18.4	96.88	2.200E-16	0.9363	0.9567	13.307	11.822
N4	18.4	27.8	31	17	24.4	629.26	2.200E-16	0.9506	N/A	11.8222	~0
N5	25	36.8	N/A	N/A	35.4	108.89	2.200E-16	N/A	N/A	N/A	N/A
P1	28	38.8	41	13	36.2	489.07	2.200E-16	N/A	0.8622	~0	25.2
P2	36	41.8	15	14	39	180.83	2.200E-16	0.8545	N/A	22.196	~0
N6	39	44.6	16	13	42.2	54.296	7.505E-11	N/A	0.717	~0	6.277
P3	42.2	49.8	14	25	44.8	488.7	2.200E-16	0.717	N/A	6.277	~0
P4	44.8	52.4	11	27	47	522.86	2.200E-16	N/A	N/A	N/A	N/A

P5	50.4	55.2	11	14	52.6	340.28	2.200E-16	N/A	0.729	~0	7.941
N7	52.6	57.6	8	17	54.2	20.699	5.573E-04	N/A	N/A	N/A	N/A
P6	55.4	61.4	14	17	58	29.522	7.782E-06	N/A	0.9345	~0	13.43
**Layer 2**											
	**SP (s)**	**EP (s)**	**N_{sb}**	**N_{be}**	**BP (s)**	**F**	**p**	**R^2_{sb}**	**R^2_{be}**	**k_{sb}**	**k_{be}**
T2	12.2	19.4	27	10	17.4	320.32	2.200E-16	0.8271	0.9111	13.35	19.436
N3	17.2	24.4	25	12	22.0	65.56	3.357E-13	N/A	N/A	N/A	N/A
N4	19.6	27.6	27	14	24.8	33.43	9.837E-07	0.732	0.8726	17.225	29.246
N5	24.2	36.2	23	38	28.6	45.47	4.767E-09	0.878	0.8594	11.105	16.051
P1	28.0	38.8	48	7	37.6	11.54	2.692E-02	N/A	N/A	N/A	N/A
P2	36.4	42.0	N/A	N/A	41.4	65.28	4.082E-13	N/A	N/A	N/A	N/A
N6	39.0	44.6	12	17	41.4	65.56	3.357E-13	0.7905	0.7706	34.206	12.836
P3	42.2	49.8	14	25	44.8	109.22	2.200E-16	N/A	N/A	N/A	N/A
P4	44.8	53.0	32	10	51.2	619.85	2.200E-16	N/A	0.7596	~0	24.68
P5	50.4	55.2	15	10	53.2	110.05	2.200E-16	0.7596	N/A	24.68	~0
N7	53.0	57.2	12	10	55.2	41.49	2.501E-08	N/A	0.8544	~0	10.937
P6	55.2	61.4	16	16	58.2	34.34	1.086E-06	0.8544	0.8434	10.937	35.271
**Layer 3**											
	**SP (s)**	**EP (s)**	**N_{sb}**	**N_{be}**	**BP (s)**	**F**	**p**	**R^2_{sb}**	**R^2_{be}**	**k_{sb}**	**k_{be}**
N4	19.0	27.2	28	13	24.6	70.73	1.902E-14	0.9556	0.9751	29.528	57.65
N5	24.2	35.8	15	44	27.2	300.56	2.200E-16	0.9695	0.9356	53.825	21.57
P1	27.4	38.8	30	27	33.4	527.20	2.200E-16	N/A	N/A	N/A	N/A
P2	36.0	41.6	16	13	39.0	147.17	2.200E-16	N/A	0.8367	~0	33.276
N6	39.0	44.6	12	17	41.4	84.51	2.200E-16	0.838	N/A	30.232	~0
P3	41.8	49.0	14	23	44.4	12.13	1.723E-02	N/A	N/A	N/A	N/A
P4	44.6	52.8	29	13	50.2	398.97	2.200E-16	N/A	0.8978	~0	23.303
P5	49.2	55.4	21	10	53.4	46.00	3.916E-09	0.9115	N/A	26.38	~0
N7	53.0	57.2	10	12	55.0	47.88	1.671E-09	N/A	0.8935	~0	15.154
P6	55.4	61.4	11	20	57.4	128.67	2.200E-16	0.8968	0.8562	16.331	20.686
**Layer 4**											
	**SP (s)**	**EP (s)**	**N_{sb}**	**N_{be}**	**BP (s)**	**F**	**p**	**R^2_{sb}**	**R^2_{be}**	**k_{sb}**	**k_{be}**
N5	23.8	36.2	19	44	27.4	7.90	7.789E-02	N/A	N/A	N/A	N/A
P1	27.8	38.8	40	16	35.8	18.79	1.293E-03	N/A	N/A	N/A	N/A
P2	36.4	41.6	12	15	38.8	19.65	6.716E-04	N/A	N/A	N/A	N/A
N6	39.0	44.6	12	17	41.2	14.64	5.878E-03	N/A	N/A	N/A	N/A
P3	41.8	50.2	11	32	44.0	583.40	2.200E-16	0.8584	N/A	35.48	~0
P4	44.8	53.6	29	16	50.4	149.84	2.200E-16	N/A	N/A	N/A	N/A
P5	50.4	54.8	12	11	52.6	34.08	7.392E-07	N/A	0.7178	~0	29.394
N7	52.6	57.2	10	16	54.6	7.64	1.135E-01	N/A	N/A	N/A	N/A
P6	55.0	61.4	12	21	57.4	469.97	2.200E-16	0.7963	0.8114	22.762	14.561
**Layer 5**											
	**SP (s)**	**EP (s)**	**N_{sb}**	**N_{be}**	**BP (s)**	**F**	**p**	**R^2_{sb}**	**R^2_{be}**	**k_{sb}**	**k_{be}**

**P1**	26.4	39.2	55	11	37.2	80.468	2.20E-16	0.9273	0.9165	15.533	9.3091
**P2**	37	41.6	12	12	39.4	52.878	6.22E-11	0.959	0.7855	17.249	12.784
**N6**	39.4	43.6	13	10	41.8	1.6315	0.8534	N/A	N/A	N/A	N/A
**P3**	41.6	49.4	11	31	43.4	22.935	1.02E-04	0.8324	0.7591	13.174	25.453
**P4**	44	52.4	27	17	49.2	120.9	2.20E-16	0.7261	0.8818	24.931	21.778
**P5**	49.6	55.4	15	16	52.6	203.33	2.20E-16	0.8818	N/A	21.778	~0
**N7**	52.4	57.4	17	10	55.8	171.24	2.20E-16	N/A	0.8954	~0	21.887
**P6**	55.4	61.4	12	20	57.8	3.4166	0.4432	N/A	N/A	N/A	N/A
**Layer 6**											
	**SP (s)**	**EP (s)**	**N_{sb}**	**N_{be}**	**BP (s)**	**F**	**p**	**R^2_{sb}**	**R^2_{be}**	**k_{sb}**	**k_{be}**
**P3**	41.6	49.4	13	27	44.2	123.68	2.200E-16	0.9431	N/A	28.98	~0
**P4**	44.4	52.6	29	13	50.0	1298.00	2.200E-16	N/A	0.9526	~0	21.832
**P5**	50.0	55.2	14	13	52.6	61.45	2.760E-12	0.9526	0.8059	21.832	12.284
**N7**	52.8	57.2	14	10	55.6	45.87	4.325E-09	0.8345	0.9446	13.487	35.161
**P6**	55.4	61.4	10	21	57.2	16.08	4.606E-03	N/A	N/A	N/A	N/A
**Layer 7**											
	**SP (s)**	**EP (s)**	**N_{sb}**	**N_{be}**	**BP (s)**	**F**	**p**	**R^2_{sb}**	**R^2_{be}**	**k_{sb}**	**k_{be}**
**P6**	54.8	61.4	15	18	57.8	100.44	2.200E-16	0.9205	0.9513	69.655	33.06

The control for H_0 rejection (*L*) over all screening tests is evaluated via ordering each breakpoint by *p*-value (Supplementary Table 2) first, then considering the sequentially ordered cases (yellow highlighting) formed by N7 of Layer 1 (L1-N7, p = 5.57E-4), P2 of Layer 4 (L4-P2, p = 6.72E-4), and P3 of Layer 5 (L5-P3, p = 1.02E-4). The reaction mechanisms that grow oxides create layers of multiple atom width (Figure 1) that approach homogeneity with time via favorable single adatom diffusion events (Supplementary Figure 25-S28), though ETEM discretely measures oxide length as fully homogenous multi-atom layers at all times. Given that time intervals are short enough to capture single adatom diffusion processes that do not produce single whole oxide layers on their own, such atomic processes would be measured as discrete stepwise oscillations in oxide layer lengths. Over longer time scales, growth rates will be measurably linear as discretization limits are overcome. However, within the limitations of these discretization and measurement error issues, residual noise around breakpoints can possibly incorrectly confirm breaks around the flat (L1-N7) and sloped (L4-P2) segments due to its characteristic rapid, non-linear fluctuations. This would cause H_0 to be falsely rejected (Type 1 Error). Thus, the L1-N7 *p*-value (5.6E-4) forms the upper boundary for the threshold *L*. In contrast, slight parabolic growth character diminishes breakpoint segment distinctiveness close (Δt < 1s) to the break (L5-P3, see R^2 and visualization of segment 'b') while not impacting actual breakpoint existence, potentially leading to false non-rejection of H_0 due to the approximation of perfect linear oxide growth (Type 2 Error). Therefore, the L5-P3 *p*-value (1.2E-4) represents a lower boundary for *L*, which will be applied conservatively to screened structural breaks (unless otherwise stated). Though differing sample sizes of 'a' and 'b' segments can slightly impact the mapping of *p*-value to *F*-test value, a *p*-value of 1.2E-4 corresponds to an *F* value of ~22.5-25 (L ~ 22.5-25) in completed tests.

Though the first criterion applied to breakpoint screening is the sup-Wald *F* test with a control (*L*), breakpoints must meet several additional criteria. Breakpoints rejected with the first criterion are highlighted in red under attributes "F" and "p" in Supplementary Table 2, including L2-P1, L3-P3, L4-N5, L4-P1, L4-N6, L4-N7, L5-N6, L5-P6, and L6-P6. The second criterion restricts deviations of fitted breakpoint times from shared initial guesses. Statistically significant breaks not relating to interlayer or concerted diffusion effects can occur in individual layers due to adjacent breaks or related considerations. Therefore, significant differences between shared, interlayer initial breakpoint guesses, and the fitted breakpoints of individual layers prevent confirmation that such breakpoints result from shared physical circumstances. Given the smallest distance between two initial breakpoint guesses (10 data points), two-sided (2) data windows, and the measurement interval $\Delta t = 0.2$ s, significant differences are estimated to exceed 0.2 x (10/2) = 1 s around either side of each break. All breaks dismissed through this criterion (red highlighting of *BP (s)* in Supplementary Table 2) deviated by much more than this threshold, including L1-N5 (7.8 s), L1-P4 (2.8 s), L2-N3 (2.6 s), L2-P2 (2.4 s), and L3-P1 (5.6 s). Screened structural breaks were also rejected for a third criterion resulting from limitations in the Chow test, which is designed to evaluate the slopes and intercepts of l^3 vs. *t* segments with significant linear slopes and intercepts. Though the test can distinguish zero slope segments from non-zero segments (L4-P3 and L6-P3), evaluating two intercept-only (slope ~ 0) segments introduces errors from non-significant slope parameters and the noise they introduce. This criterion can also be related to, and considered to partially address, the Type 1 error concerns mentioned previously. Breaks L2-P3 and L4-P4 (red highlighting of R^2 in Supplementary Table 2) are omitted from structural break consideration due to this criterion.

Screened structural breaks applied univariate regressions to single-layer growth vs. *t*, inferring fitted breaks at slightly different breakpoints over multiple consecutive layers. To inferentially determine whether these are linked to a common reaction process, such as concerted interlayer Cu diffusion over consecutive oxide layers, a multivariate time series model is applied to sets of screened breaks that must reject H_0 over multiple consecutive layers simultaneously:

$$l_{i,t}^3 = \left(\frac{dl^3}{dt}\right)_i t + l_{i,t=0}^3 + \delta_{t=break} + \varepsilon_{i,t} \qquad \text{(Supplementary Equation 5)}$$

In this model, time series and data windows shared over consecutive layers are applied to concerted diffusion breaks at P6 (*i* = Layers 1, 2, 3, and 4) and interlayer nucleation effect breaks at N4 (*i* = Layers 1, 2, and 3). These series (L1-N4, L2-N4, L3-N4 and L1-P6, L2-P6, L3-P6, L4-P6) form dependent variable (l^3) matrices, rather than vectors, that are regressed versus time. Otherwise maintaining univariate model notation, a time-constant dummy variable (δ) fits a single breakpoint to all layers simultaneously. Time series and data windows were selected based on screening results, namely to produce probable consecutive breaks and increased (resampled) segment sizes.

With matching Shapiro-Wilk (SW) test results, Supplementary Figure 17 evaluates univariate regression residual normality for P4 and N6 – and thus sup-Wald *F* test applicability – via Quantile-Quantile (Q-Q) plots, investigating individual oxide layers with dummy variables placed at refitted breakpoints (Supplementary Table 3). The null hypotheses (H_0) of normality for completed SW tests cannot be rejected for any tested regression, neither for particular concerted diffusion (L4-P6, $p = 0.061$) nor interlayer nucleation (L1-N4, $p = 0.848$) event examples, when applying a strict criterion for rejecting normality ($p < 0.01$). Even in concerted

diffusion cases closer to rejecting H_0, small deviations from normality would not likely affect comparisons across oxide layers with similar features. Q-Q plots (Supplementary Figure 17) indicate that the primary contributions to non-normality are pronounced asymmetric negative deviations, which would occur across all layers with shared sharp sup-Wald *F* test suprema (Supplementary Figure 18) and common discretization or sampling issues associated with 7th layer affected growth rates (Supplementary Figure 16). Thus, comparisons between layers would likely (at least partially) cancel out effects of normality deviations.[38]

Supplementary Table 3 indicates that though linear fits are similarly strong across all refitted univariate results, linear models encompassing breakpoints (*M*) are universally better than those for data segments (*sb*, *be*), while models with dummy variables (*M*+*D* model) universally improve over *L* models. General Wald *F*-tests comparing the goodness-of-fit of *M* and *M*+*D* models favor adding dummy variables, which have low coefficient *p*-values, at breakpoints unanimously. Concerns of the cubic model scaling assumption (l^3 vs. *t*) and the effects of approximating growing oxide rate-order kinetics are mitigated via scale-invariant Log-likelihood ratio (LR) tests mirroring these Wald tests, the results of which further support breakpoint use.[35, 36]

To reconcile resampled univariate and multivariate (Total) models, Supplementary Figure 18 plots the probability sample spaces (sup-Wald *F*-test statistic vs. time) for individual oxide layers, the overlap of which visualizes the coordinated actions of multiple oxide layers for N4 and P6 breakpoints. Probability maxima for individual layers during concerted diffusion (P6) strongly overlap and highly exceed the *L* threshold ($F \sim 22$-25). Sup-Wald *F* tests over individual and combined layer models show that P6 has very similar fitted breakpoints (BP) set by highly significant dummy variables (*p*(D)), regardless of whether univariate or multivariate models are considered (Supplementary Table 3). Goodness-of-fit tests (*M* vs. *M*+*D* Wald, LR) also select the multivariate P6 model over its univariate analog and indicate significance beyond *L* relative to *p* values.

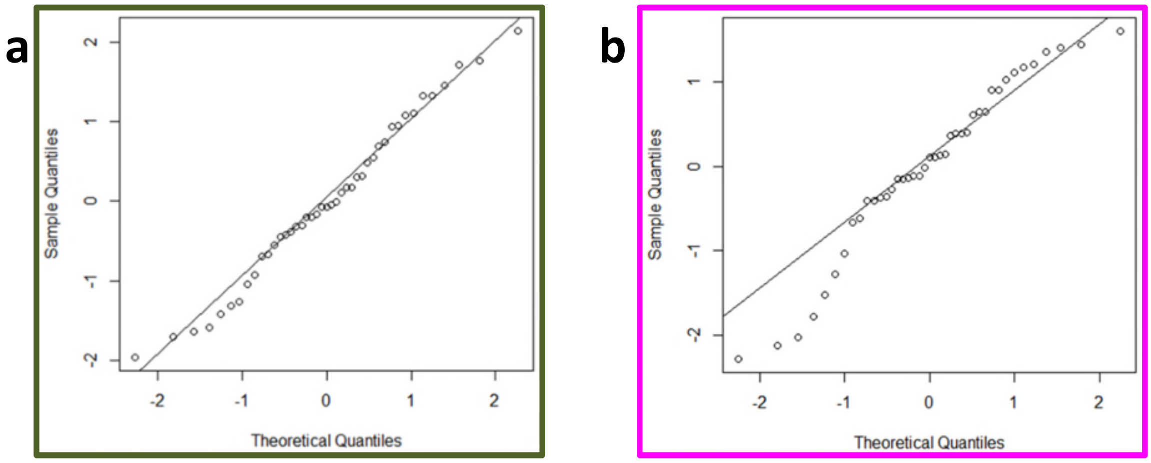

Supplementary Figure 17 | Q-Q plots of multivariate regression standardized residuals

Q-Q plots of multivariate regression standardized residuals validating regressions on **(a)** L1-N4 (green outline) and **(b)** L4-P6 (pink outline) systems, with dummy variables accounting for calculated structural breaks. Shapiro-Wilk test *p*-values for L1-N4 ($p = 0.848$) and L4-P6 ($p =$

0.061) characterize all other systems sharing a tested single break in time that were tested for normality.

Supplementary Table 3 | Multivariate analysis summary of P6 and N4 structural breaks

R^2 for segments before (sb) and after (be) each shared break of each layer (M), sample sizes of each segment (N) after fitted breaks, R^2 values of single linear models neglecting breaks [R^2(M)], R^2 of linear models including breaks [R^2(M+D)], *p* values of dummy variables (D) modeling breaks [*p*(D)], fitted breaks (BP), *F* statistics [*F*(Wald)] and matching *p* values [*p*(Wald)] of general Wald tests comparing M and M+D models, and chi-squared [χ^2(LR)] and matching *p* values [*p*(LR)] of corresponding log-likelihood ratio tests. Test results are presented for the individual layer and multivariate (Total) models.

**P6**			**t(start)**	53	**t(end)**	61.4	**N**	43	x 4 =	172		
	**R^2_{sb}**	**R^2_{be}**	**N_{sb}**	**N_{be}**	**R^2(M)**	**R^2(M+D)**	***p*(D)**	**BP (s)**	***F*(Wald)**	***p*(Wald)**	**χ2(LR)**	***p*(LR)**
**L1**	0.8862	0.9206	24	19	0.9417	0.9653	3.60E-06	57.6	28.857	3.60E-06	23.356	1.35E-06
**L2**	0.8072	0.8466	22	21	0.8961	0.9333	1.69E-05	57.2	23.897	1.69E-05	20.141	7.19E-06
**L3**	0.7425	0.8045	22	21	0.891	0.976	6.23E-15	57.2	146.1	6.23E-15	66.109	4.27E-16
**L4**	0.9031	0.8114	22	21	0.9081	0.9897	2.00E-16	57.2	323.92	2.20E-16	94.946	2.20E-16
**Total**			88	84	0.9265	0.9857	5.40E-16	57.2	170.16	5.40E-16	71.335	2.20E-16
**N4**			**t(start)**	21.2	**t(end)**	29.2	**N**	41	x 3 =	123		
	**R^2_{sb}**	**R^2_{be}**	**N_{sb}**	**N_{be}**	**R^2(M)**	**R^2(M+D)**	***p*(D)**	**BP (s)**	***F*(Wald)**	***p*(Wald)**	**χ2(LR)**	***p*(LR)**
**L1**	0.9046	N/A	19	22	0.9057	0.9671	1.99E-10	25	73.683	1.99E-10	44.201	2.96E-11
**L2**	N/A	0.8562	17	24	0.9179	0.9306	0.00711	24.6	8.0947	0.007114	7.9176	0.004896
**L3**	0.9576	0.9628	21	20	0.9811	0.9852	0.00141	25.4	11.855	0.001415	11.133	0.000848
**Total**			57	66	0.9732	0.9851	1.52E-06	25	32.36	1.52E-06	25.26	5.02E-07

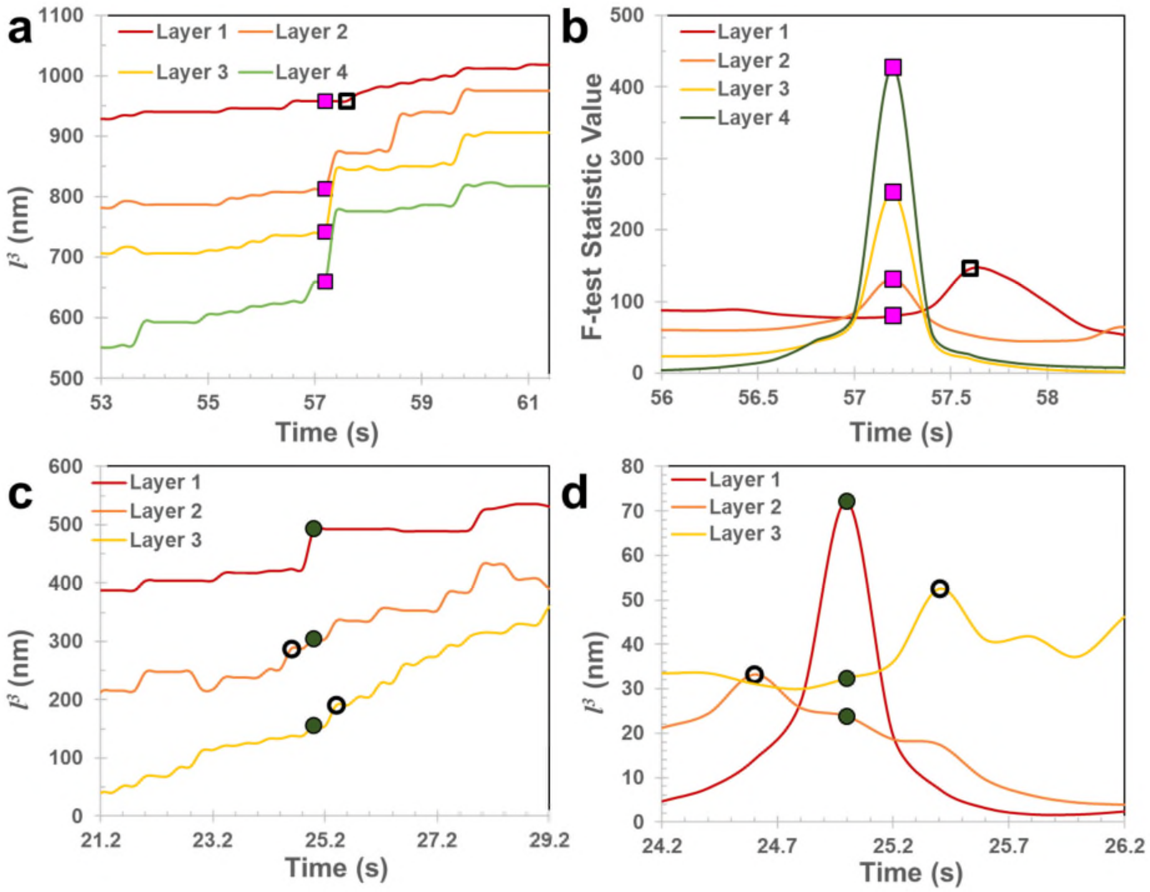

Supplementary Figure 18 | Overlapping probability sample spaces of each layer in multivariate analysis.

Fitted l^3 *vs. t* structural breaks for P6 (**a**) and matching sup-Wald F test statistics over data windows (**b**), with matching results for N4 (c and d, respectively). Unfilled and filled markers indicate the single-layer and multi-layer F test maxima determining breakpoints for respective models.

In contrast, univariate models for N4 have more distinct breakpoints relative to their multivariate analog (25.0 s) than P6 models (Supplementary Figure 17, Supplementary Table 3). Given the *F*-test statistic (L ~ 22-25) and *p*-value criteria ($p < 1.2E-4$) for screened results, univariate model dummy variable *p*-values, sup-*F* Wald probability space overlap (univariate models with a multivariable breakpoint), and *M* vs. *M+D* Wald tests show either no or ambiguous significance for L2-N4 and L3-N4 layers. Nevertheless, the N4 multivariate model ("Total") confirms the significance of these statistical premises and matching LR results with a single breakpoint in Supplementary Table 3 via results satisfying *L*. This conclusion validates the multivariate approach for interlayer nucleation events, and illustrates the usefulness of reviewing multiple individual local probability space maxima with sup-*F* testing.

Correlating Statistical Results with Experimental Observations

Statistically assessed breakpoints and matching reactions can be correlated to experimental ETEM observations found in Supplementary Movie 1. For example, intralayer diffusion for Layer #2 (T2, Supplementary Figure 16) at 16.6 s coincides with a developing dark interface on top of the 1st grown oxide layer, which was located beyond the growth front of the new 2nd layer. Immediately following 16.6 s, this dark underlying interface was replaced by the 2nd oxide layer rapidly growing over it. This dark interface was an underlying reconstructed surface. Thus, this intralayer diffusion process and breakpoint correlate to the threshold at which a reconstructed oxide surface transitions to its growth phase, namely with the supply of Cu sourced from the current oxide layer.

Concerning breakpoint N4 (25.0 s), Supplementary Movie 1 depicts an interconnected, sequential set of mechanisms over multiple individual layers that correspond to their individual breakpoints as well. As the 4th oxide layer nucleates, the 3rd and 2nd layer growth fronts simultaneously begin to retreat and expand (24.6 s) with respect to one another, respectively. Here, the 3rd layer growth front approaches the 2nd layer step edge, implying an Ehrlich-Schwöbel effect may be responsible for concerted layer movement, and that the 3rd layer supplies Cu to form the 4th layer. Subsequently, the 1st oxide layer expands (25.0 s), receiving Cu sourced either from the expanded 2nd layer or the underlying substrate. Finally, the 3rd layer growth front expands (25.4 s) relative to the 2nd layer front via Cu transfer from the first two layers. Oxide layer expansion order (2nd, 1st, 3rd) directly correlates with fitted individual oxide layer breakpoint order (24.6, 25.0, 25.4 s), physically supporting and linking the univariate and multivariate models of this interlayer nucleation event.

The concerted diffusion event at 57.4 s in Supplementary Movie 1 (P6, Supplementary Figure 12) correlates with a step self-adjustment of the Cu_2O island top. Originally, the island top was not flat and shows zig-zag facets of $Cu_2O(110)$ (Figure 1(a)). During layer-by-layer growth of the island, new layer fronts 'retreat' when their growth nears the edge of the previous layer (triangles in Figure 1(f)), implying an Ehrlich-Schwöbel effect. Each new layer grows and stops at a similar length, while layers continue to form until the oxide island top observes a flat (100) orientation (Figure 1(d)). These events can also occur when previous layers all undergo concerted growth to adjust island top shape in a single step process, physically substantiating breakpoint agreement over univariate and multivariate P6 models.

Software Specifications

Lastly, note that *p*-value results of 2.2E-16 achieved in this study are invariantly assigned to large *F*-test statistics due to limitations in the calculator precision of applied R functions. The *Fstats* function in the "strucchange" R package calculates *F*-test statistics,[36] while *M* vs. *M+D* Wald and LR tests are done via *waldtest* and *lrtest* functions in the "lmtest" R package, respectively.[39]

Supplementary Note 5: DFT results on gas/solid interfacial energies

The surface of $Cu_2O(100)$ has two terminations, namely Cu-terminated (termed '100, Cu') and O-terminated ('100, O'), while the surface of $Cu_2O(110)$ also has two terminations, namely Cu-terminated ('110, Cu') and Cu-O terminated ('110, Cu-O'). This section features the calculated gas-solid interface energies of structures encountered during the growth of isolated, epitaxial Cu_2O oxide islands (each formed relative to Cu_2O unit cell dimensions) on these four different Cu_2O surface orientations and terminations. For each trend plotted in Supplementary Figure 21, the maximum number of simulated oxide layers equals the minimum number of simulated layers needed to periodically repeat the symmetry of the grown oxide island.

Supplementary Figure 19-S22 respectively visualize isolated, epitaxial Cu_2O oxide island growth processes on Cu terminated $Cu_2O(100)$, O terminated $Cu_2O(100)$, Cu terminated $Cu_2O(110)$, and Cu-O terminated $Cu_2O(110)$. Supplementary Table 4 shows the calculated surface energies of each visualization. Supplementary Figure 23 plots the summary of the DFT surface energies. All visualizations of the simulation results below are produced by VESTA[40].

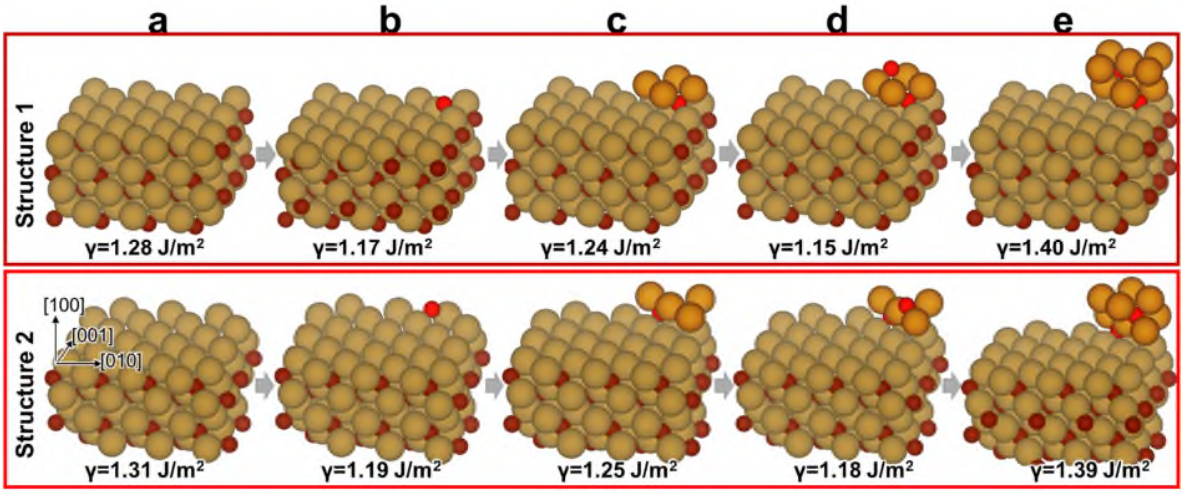

Supplementary Figure 19 | γ of Cu-terminated $Cu_2O(100)$ surface with increasing Cu_xO_y surface units.

Isolated, epitaxial $Cu_2O(100)$ oxide island growth on a Cu terminated $Cu_2O(100)$ surface, starting from a (**a**) flat surface (*i*, Figure 2) and proceeding to (**b**) 0.5 (*ii*, Figure 2), (**c**) 1.0 (*iii*, Figure 2), (**d**) 1.5, and (**e**) 2.0 added Cu_xO_y surface units growth. In structure 1, the interface has O on the 0.5 layer bonded to a 4 atom "square" Cu sheet. In structure "2", the interface has O on the 1.5 layer bonded to a 4 atom "square" Cu sheet.

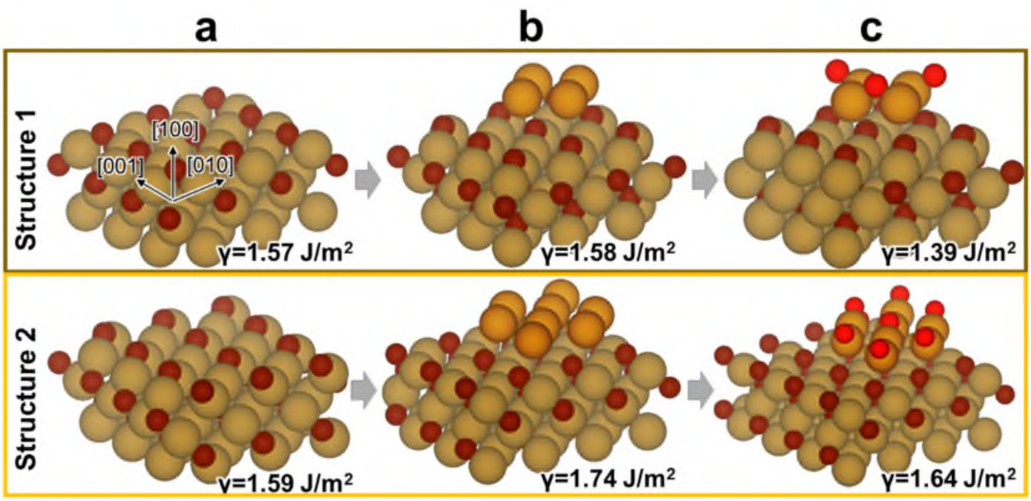

Supplementary Figure 20 | γ of O-terminated $Cu_2O(100)$ surface with increasing Cu_xO_y surface units.

Isolated, epitaxial $Cu_2O(100)$ oxide island growth on an O terminated $Cu_2O(100)$ surface, starting from (**a**) flat surface (*i*, Figure 2) and proceeding to (**b**) 0.5 (*ii*, Figure 2) and (**c**) 1.0 (*iii*, Figure 2) layer growth. In structure "1", the interface has O on the flat surface directly exposed to the growing 0.5 Cu layer. In structure "2", the interface has Cu on the flat surface directly exposed to this growing layer.

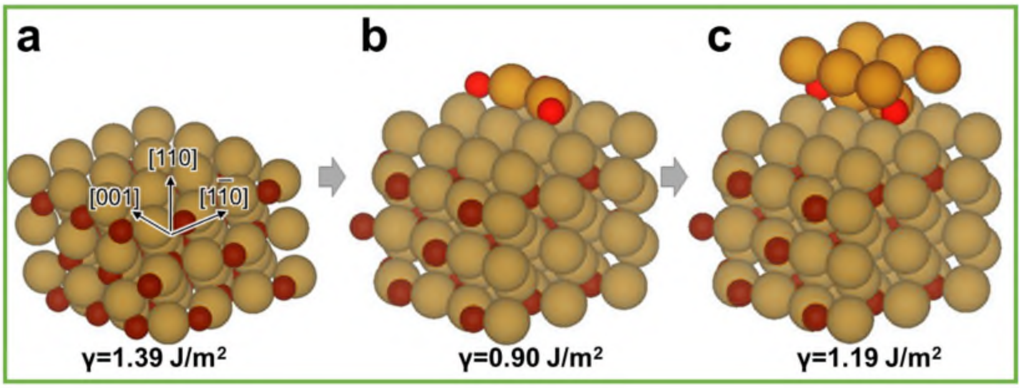

Supplementary Figure 21 | γ of Cu-terminated $Cu_2O(110)$ surface with increasing Cu_xO_y surface units

Isolated, epitaxial $Cu_2O(110)$ oxide island growth on a Cu terminated $Cu_2O(110)$ surface, starting from (**a**) flat surface (*i*, Figure 2) and proceeding to (**b**) 0.5 (*ii*, Figure 2) and (**c**) 1.0 (*iii*, Figure 2) layer growth.

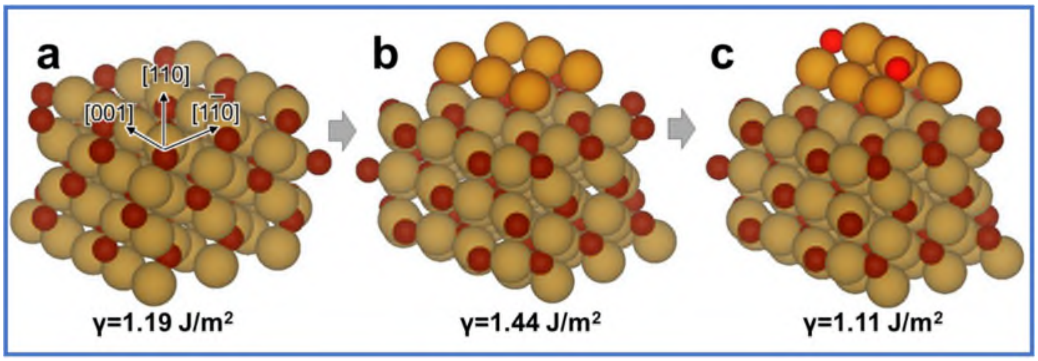

Supplementary Figure 22 | γ of Cu-O terminated $Cu_2O(110)$ surface with increasing Cu_xO_y surface units

Isolated, epitaxial $Cu_2O(110)$ oxide island growth on a Cu-O terminated $Cu_2O(110)$ surface, starting from (**a**) flat surface (*i*, Figure 2) and proceeding to (**b**) 0.5 (*ii*, Figure 2) and (**c**) 1.0 (*iii*, Figure 2) layer growth.

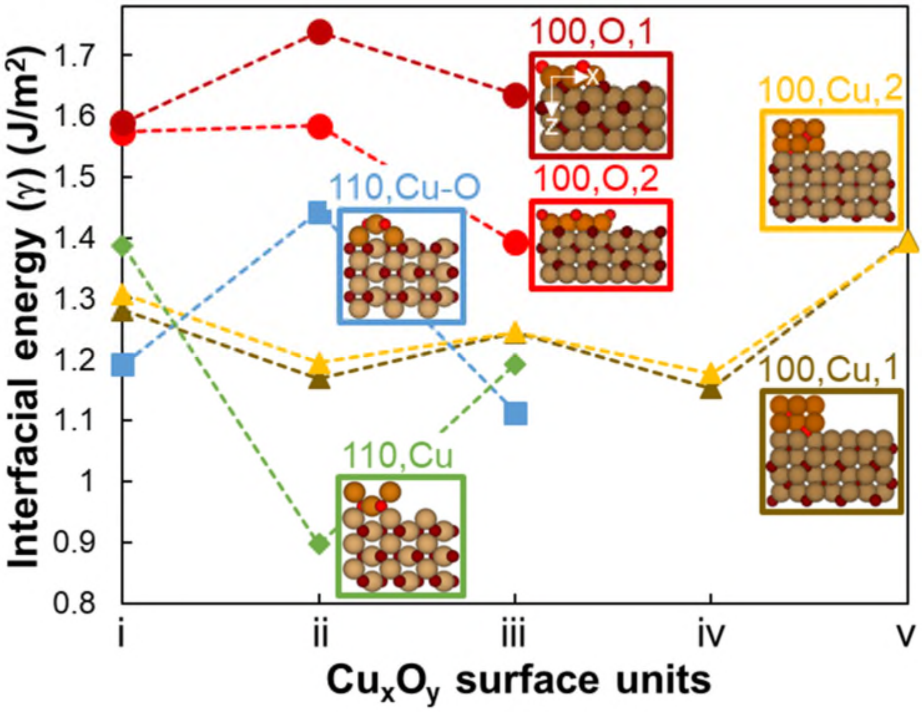

Supplementary Figure 23 | Plot of the calculated γ with increasing Cu_xO_y surface units.

Interfacial energies of the most favorable half-layer and integer layer structures for Cu terminated $Cu_2O(110)$ ('110, Cu', green, diamond), Cu-O terminated $Cu_2O(110)$ ('110, Cu-O', blue, square), O terminated $Cu_2O(100)$ ('100, O', red, circle), and Cu terminated $Cu_2O(100)$ ('100, Cu', yellow, triangle) surfaces. '100, O' surfaces in the *z*-direction can be Cu (dark red, "1") or O (light red, "2") terminated in the *x*-direction. '100, Cu' surfaces can be Cu (dark yellow, "1") or Cu-O (light yellow, "2") terminated nearest to vacuum facing the *x*-direction.

Supplementary Table 4 | Summary of interfacial energies of grown Cu_xO_y surface unit calculations described in Supplementary Figures 19-22.

Figure	Orientation	Termination	Label	Image	Oxide Layer (#)	γ (J/m²)
Supplementary Figure 19	(100)	Cu	1	a	0.0	1.28
				b	0.5	1.17
				c	1.0	1.24
				d	1.5	1.15
				e	2.0	1.40
	(100)	Cu	2	a	0.0	1.31
				b	0.5	1.19
				c	1.0	1.25
				d	1.5	1.18
				e	2.0	1.39
Supplementary Figure 20	(100)	O	1	a	0.0	1.57
				b	0.5	1.58
				c	1.0	1.39
	(100)	O	2	a	0.0	1.59
				b	0.5	1.74
				c	1.0	1.64
Supplementary Figure 21	(110)	Cu	-	a	0.0	1.39
			-	b	0.5	0.90
			-	c	1.0	1.19
Supplementary Figure 22	(110)	Cu-O	-	a	0.0	1.19
			-	b	0.5	1.44
			-	c	1.0	1.11

Supplementary Note 6: DFT results for adsorption sites on Cu_2O surfaces during oxide growth

From the analysis performed in Note 3, $Cu_2O(110)$ surfaces appear to be more energetically favorable than their $Cu_2O(100)$ analogs, in addition to observing complementary oscillatory behavior favoring the exposure of Cu-O terminated island interfaces. To verify that this favorability is maintained over single adatom adsorption events, per adatom adsorption events constructing the first island half-layer of each $Cu_2O(110)$ surface termination are investigated. Supplementary Figure 24 depicts candidates for adsorption states on Cu terminated $Cu_2O(110)$, namely the first, second, third, and fourth adatoms forming the first island half-layer (0.5 layer or *ii*, Figure 2). Correspondingly, Supplementary Figure 25 shows candidate adsorption states of the first, second, third, fourth, and fifth adatoms contributing to the 0.5 layer (*ii*, Figure 2) of Cu-O terminated $Cu_2O(110)$. Supplementary Table 5 outlines the adsorption energies of each of these structures and labels them accordingly. Supplementary Figure 26 plots the summary of the most favorable adatom adsorption state calculation results.

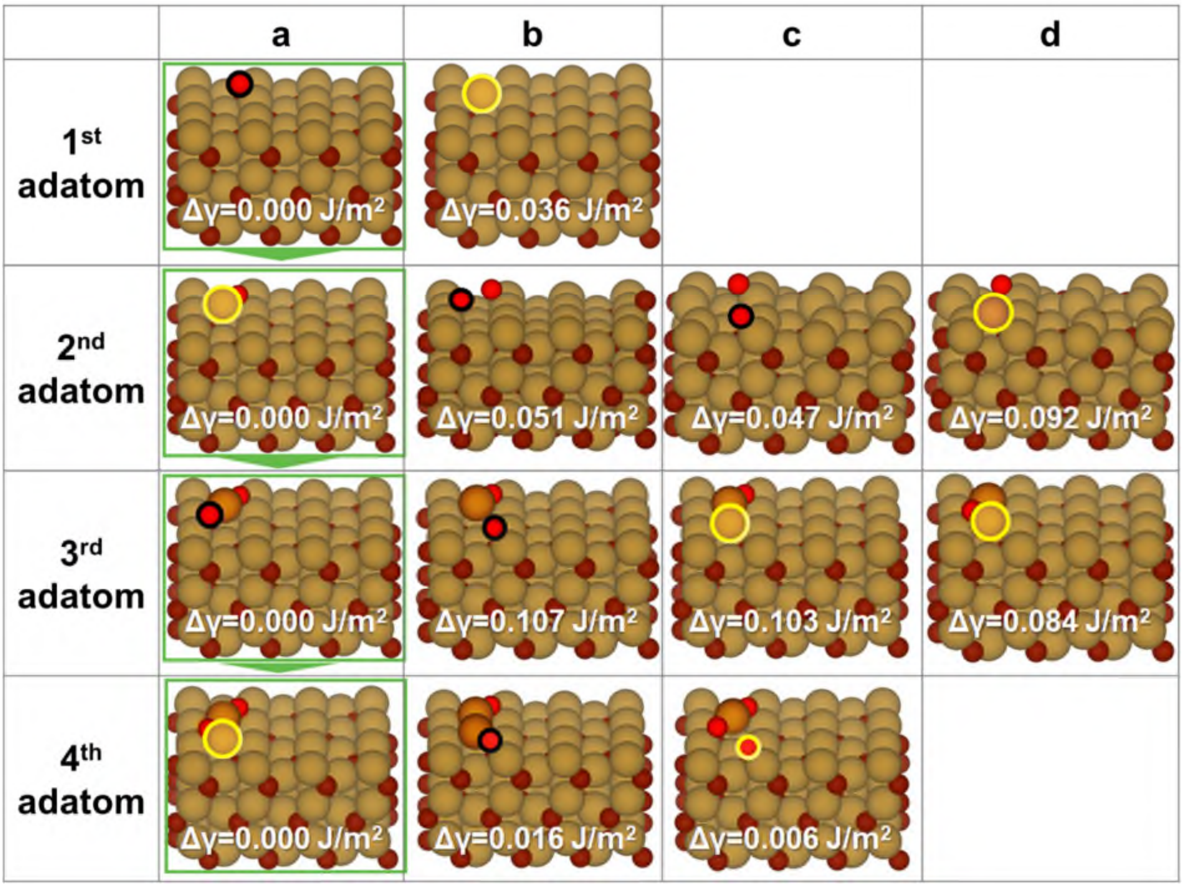

Supplementary Figure 24 | 1st-4th adatom adsorption states on Cu terminated $Cu_2O(110)$ surface.

Candidate adatom adsorption states on the Cu terminated $Cu_2O(110)$ surface, which contributes the 1st -4th adatoms to the 0.5 layer (*ii*, Figure 2) of this structure. The 2nd-4th adatom positions

are tested based on the most favorable previous (1st-3rd) adatom states highlighted in green boxes. Newly added O adatoms are outlined in black, while matching Cu adatoms are outlined in yellow. The Δγ of each adatom configuration is calculated relative to the most favorable configuration at a given number of adatoms.

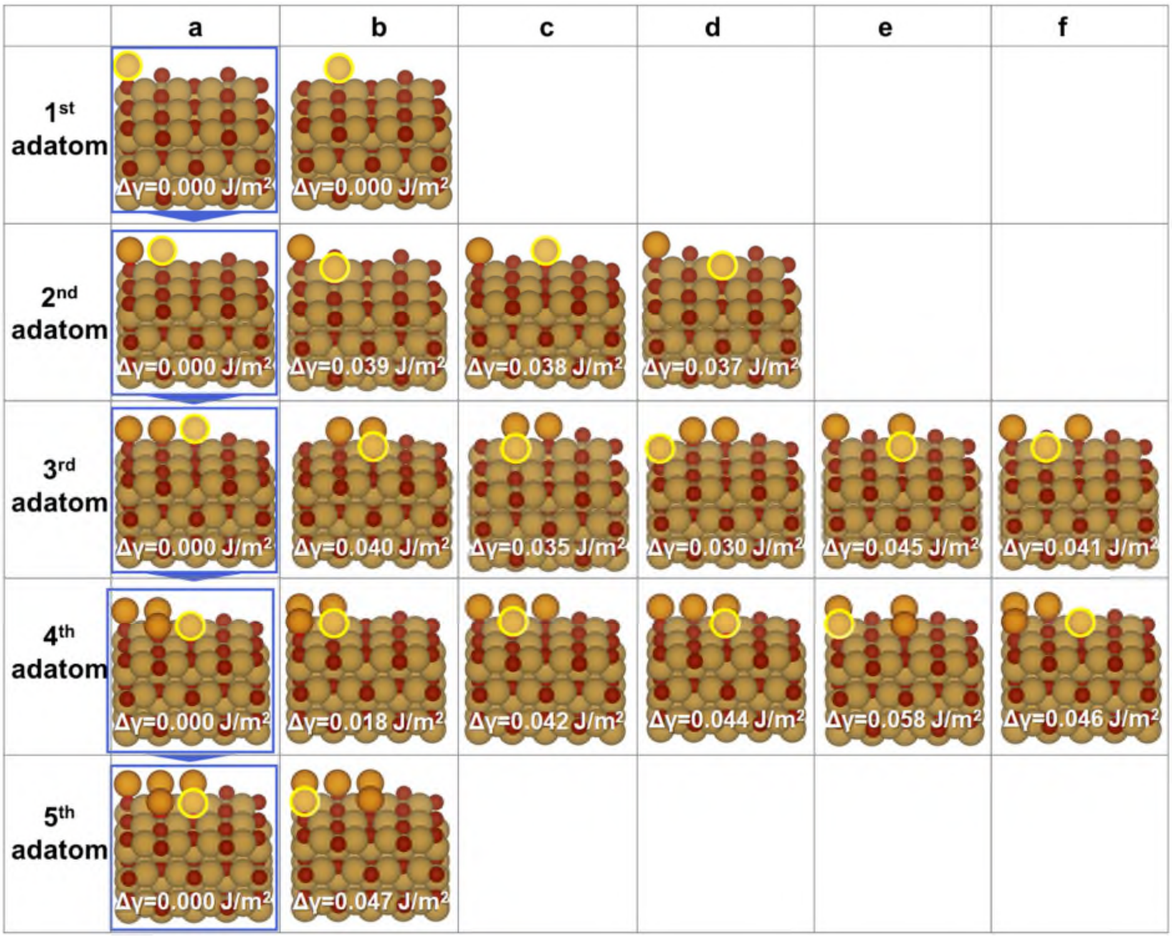

Supplementary Figure 25 | 1st-5th adatom adsorption states on Cu-O terminated Cu_2O(110) surface.

Candidate adatom adsorption states on the Cu-O terminated Cu_2O(110) surface, which contributes the first five Cu adatoms (newest added highlighted in yellow) to the 0.5 layer (*ii*, Figure 2) of this structure. The 2nd-5th adatom positions are tested based on the most favorable previous (1st-4th) adatom states highlighted in blue boxes. The Δγ of each adatom configuration is calculated relative to the most favorable configuration at a given number of adatoms.

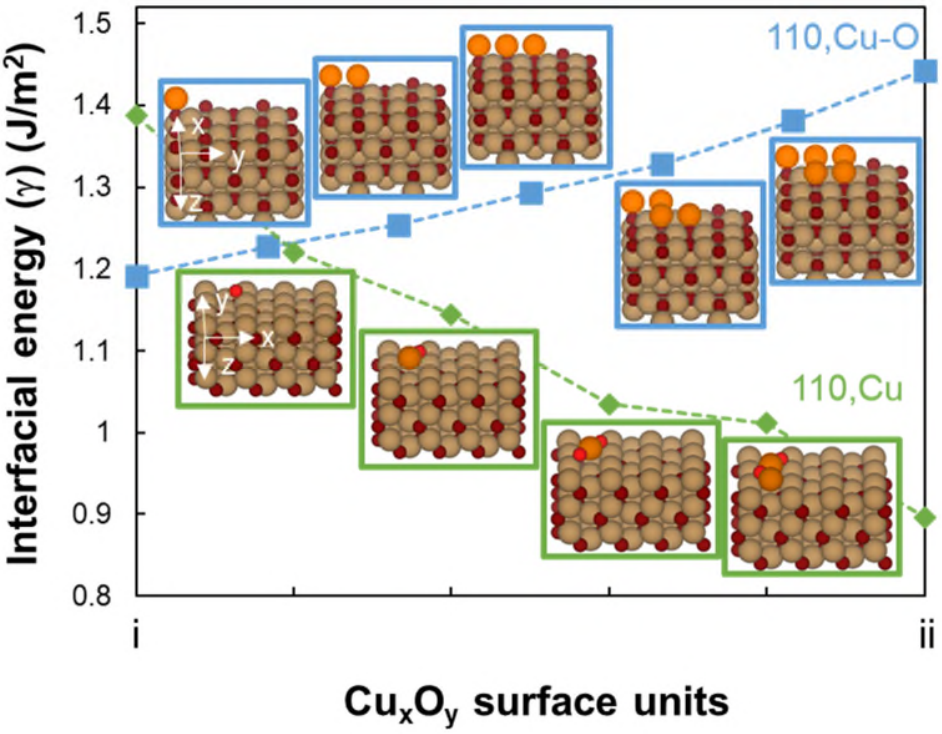

Supplementary Figure 26 | Most favorable gas-solid interface energies of intermediate structures observed while growing Cu_xO_y structure (*ii*, Figure 2) on $Cu_2O(110)$.

Most favorable gas-solid interface energies of intermediate structures observed while growing the 0.5 layer (*ii*, Figure 2) of Cu-O terminated $Cu_2O(110)$ (blue, square) and Cu terminated $Cu_2O(110)$ (green, diamond) per adatom. Each pictured structure (inset) nearest to its corresponding surface energy features a newly added atom, with Cu adatoms marked with brighter colors.

Supplementary Table 5 | Summary of relative per adatom surface adsorption energies described in Supplementary Figures 24-25.

The Δγ of each adatom configuration is calculated relative to the most favorable configuration at a given number of adatoms.

Surface	Adatom #	Candidate	Adatom configuration	Δγ (J/m²)	γ (J/m²)
**(110),Cu** Supplementary Figure 24	1st	**a - O**	**O**	**0.000**	**1.221**
		b – Cu	Cu	0.036	
	2nd	**a – Cu**	**O-Cu**	**0.000**	**1.145**
		b – O	O & O	0.051	
		c – O	O, O	0.047	
		d – Cu	O, Cu	0.092	
	3rd	**a – O**	**O-Cu-O**	**0.000**	**1.035**
		b – O	O-Cu & O	0.107	
		c – Cu	O-Cu & Cu	0.103	
		d – Cu	Cu-O-Cu	0.084	
	4th	**a – Cu**	**O-Cu-O-Cu**	**0.000**	**1.012**
		b – O	O-Cu & O-Cu	0.016	
		c – O	O-Cu-O & O	0.006	
**(110),Cu-O** Supplementary Figure 25	1st	**a – Cu**	**Edge of the substrate**	**0.000**	**1.227**
		b – Cu	Center of the substrate	0.000	
	2nd	**a – Cu**	**Nearest neighbor site**	**0.000**	**1.254**
		b – Cu	Second nearest neighbor site	0.039	
		c – Cu	Third nearest neighbor site	0.038	
		d – Cu	Fourth nearest neighbor site	0.037	
	3rd	**a – Cu**	**linear Cu-Cu-Cu**	**0.000**	**1.293**
		b – Cu	bent Cu-Cu-Cu	0.040	
		c – Cu	bent Cu-Cu-Cu	0.035	
		d – Cu	Cu & Cu-Cu (within two nearest neighbor sites of one another)	0.030	
		e – Cu	Cu & Cu-Cu (within three nearest neighbor sites of one another)	0.045	
		f – Cu	three separate Cu adatoms	0.041	
	4th	**a – Cu**	**bent Cu-Cu-Cu-Cu**	**0.000**	**1.328**
		b – Cu	Square Cu-Cu-Cu-Cu	0.018	
		c – Cu	triangular Cu-Cu-Cu-Cu	0.042	
		d – Cu	L-shaped Cu-Cu-Cu-Cu	0.044	
		e – Cu	Cu-Cu and Cu-Cu	0.058	
		f – Cu	Cu-Cu-Cu and Cu	0.036	
	5th	**a – Cu**	**Corner Cu missing from the 0.5 layer structure**	**0.000**	**1.381**
		b – Cu	edge Cu missing from the 0.5 layer structure	0.047	

Supplementary Note 7: DFT results on Cu and O diffusion on Cu_2O surfaces

From the analysis performed in Supplemental Note 3, Cu_2O(110) oxide island interfaces are confirmed to be more energetically favorable than their Cu_2O(100) analogs at the level of single adatom adsorption, supporting the corresponding analysis of adsorbed surface layers assessed in Supplemental Note 6. However, the reaction mechanisms linking these adsorption states and related states – which review the diffusion of Cu atoms, O atoms, and atomic clusters to form oxide island layers – have not yet been performed to confirm the relative favorability of oxide island layer growth on Cu_2O(110) surfaces over Cu_2O(100) surfaces. Supplementary Figure 25 reviews candidate O diffusion processes used to form the first half-layer (*ii,* Figure 2) of Cu_2O and the diffusion of Cu to form the second half-layer (*iii,* Figure 2) of Cu_2O on Cu_2O(100) surfaces (Cu termination). Supplementary Figure 26 reviews candidates for the diffusion of Cu on Cu-O terminated Cu_2O(110). Supplementary Figure 27 reviews the Cu, O, and atomic cluster diffusion processes constituting the unique reactions needed to form the 0.5 layer (*ii,* Figure 2) of Cu-terminated Cu_2O(110). Supplementary Table 6 outlines the diffusion barriers associated with each of these processes. Supplementary Figure 28 summarized the most favorable Cu and O diffusion processes during Cu_2O growth.

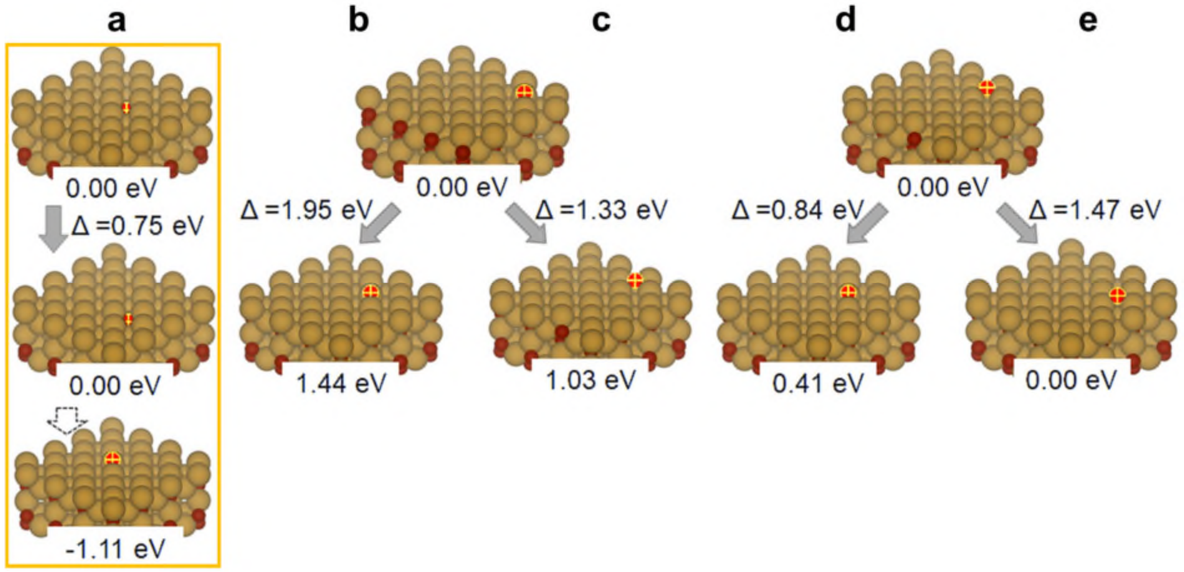

Supplementary Figure 27 | Adatom diffusion events on Cu-terminated Cu_2O(100) forming the 0.5 layer (*ii,* Figure 2) Cu_2O structure.

The starting and ending structures of the diffusion events evaluated are shown, the diffusing atoms are highlighted with yellow crosses, and the most favorable candidate of adatom event is highlighted with a yellow box. The diffusion paths evaluated are formed from linear trajectories connecting the two terminal structures. These diffusion events correspond to the adsorption state calculations shown in Supplementary Figure 17. Diffusion barriers (Δ) with adsorption state energies are connected by filled arrows, while adsorption state energy differences in isolation are connected by dashed arrows.

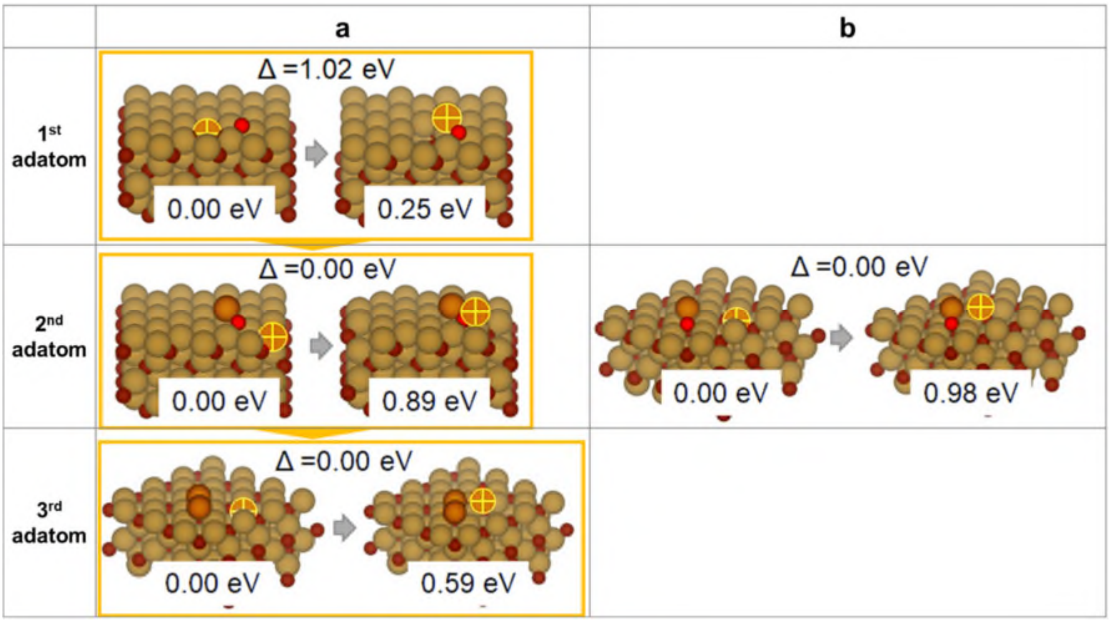

Supplementary Figure 28 | Adatom diffusion events on Cu-terminated Cu_2O(100) forming the 1.0 layer (*iii*, Figure 2) Cu_2O structures.

The starting and ending structures of the diffusion events evaluated are shown, the diffusing atoms are highlighted with yellow crosses, and the most favorable candidate for each stage of the adatom event is highlighted with a yellow box. The diffusion paths evaluated are formed from linear trajectories connecting the two terminal structures. These diffusion events correspond to the adsorption state calculations shown in Supplementary Figure 17. Diffusion barriers (Δ) with adsorption state energies are connected by filled arrows.

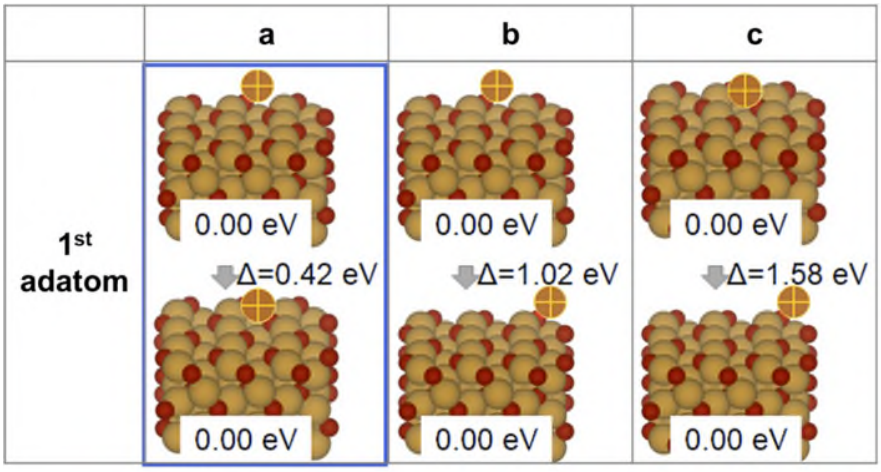

Supplementary Figure 29 | Cu adatom diffusion events on Cu-O terminated Cu_2O(110) forming the 0.5 layer (*ii*, Figure 2) Cu_2O structure.

3 candidate Cu diffusion events on the flat Cu-O terminated Cu_2O(110) surface are tested, which can contribute the first adatom to the 0.5 layer (*ii*, Figure 2) of this structure via (a) in-channel diffusion, (b) cross-channel diffusion on matching sites, and (c) cross-channel diffusion on

opposing sites. The starting and ending structures of the diffusion events evaluated are shown, the diffusion paths evaluated are formed from linear trajectories connecting each pair of terminal structures, and highlighted atoms indicate which atoms diffuse during the events. The most favorable candidate for each stage of the adatom event is highlighted with a blue box. These diffusion events correspond to the adsorption state calculations shown in Supplementary Figure 20.

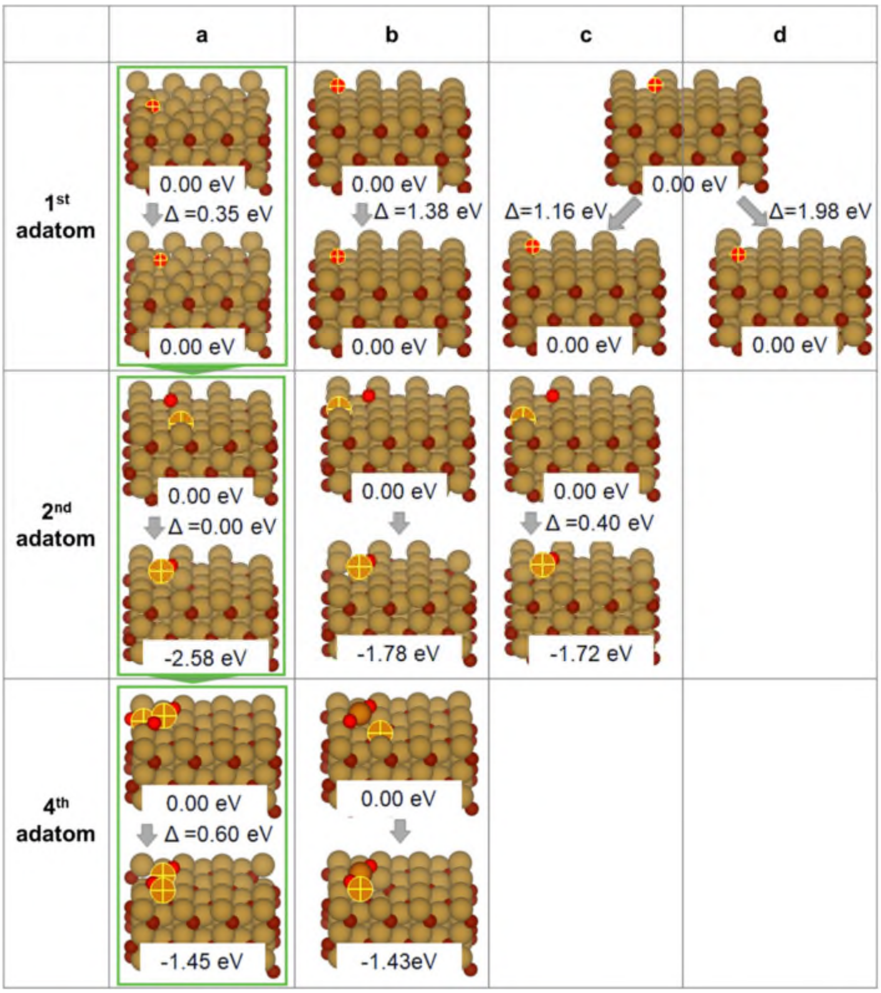

Supplementary Figure 30 | Adatom diffusion events on Cu terminated Cu_2O(110) to form the 0.5 layer (*ii,* Figure 2) Cu_2O structure.

The starting and ending structures of the diffusion events evaluated are shown, the diffusing atoms are highlighted with yellow crosses, and the most favorable candidate for each stage of the adatom event is highlighted with a green box. These diffusion events correspond to the adsorption state calculations shown in Supplementary Figure 19.

For the 1st adatom calculations, (**a**) subsurface to surface diffusion, (**b**) in-channel diffusion, (**c**) cross-channel diffusion over adjacent (nearest possible) sites, and (**d**) cross-channel diffusion over diagonal (next nearest possible) sites are calculated. Diffusion paths are formed from

quadratic (**b** & **c**) / linear (**a** & **d**) trajectories connecting each pair of terminal structures. Note in **b** & **c**, the paths are concave parabolas with O diffusion through the interiors of channels, as the linear equivalents of the trajectories tested on these structures had unphysical path energies. Also note that the trajectory taken by (**a**) spans the initial (or an equivalently bonded) structure of the next event (2nd adatom) forming the 0.5 layer (*ii*, Figure 2) of this structure. Thus, an intermediate state obtainable by (**a**), which can be performed symmetrically across the surface channel, initializes (**b**). As a reaction mechanism, **(a)** represents a chemisorbed state participating in the only necessary rate-limiting reaction step forming the 0.5 layer (*ii*, Figure 2). In an experimental context, this surface diffusion step would both lead and constitute a limit to oxide layer growth and – given its presence – the development of intermediate, reconstructed copper oxide structures.

For the 2nd adatom calculations, (**a**) second nearest neighbor diffusion, (**b**) nearest neighbor diffusion, and (**c**) third nearest neighbor diffusion are tested, while the diffusion paths evaluated are formed from linear trajectories connecting each of two terminal structures.

For the 4th adatom calculations, (**a**) concerted diffusion and (**b**) in-channel diffusion are tested, while the diffusion paths evaluated are formed from linear trajectories connecting the two terminal structures. Note that in **(a)**, the Cu atom forming the surface Cu layer diffuses upward to the height of adsorbed O atoms, while the Cu already at the adsorbed O height diffuses in-channel. In this concerted event, both Cu adatoms diffuse simultaneously as an effective cluster. Sets of endpoint adsorption sites in both **(a)** and **(b)** were selected based on the most favorable corresponding results shown in Supplementary Figure 22. Supplementary Figure 24 shows that the surface energies of the most favorable adatom structures monotonically decrease with the number of adatoms adsorbed to the Cu terminated $Cu_2O(110)$ surface. Thus, both **(a)** and **(b)** final endpoints would readily form, notwithstanding consideration of energy barriers.

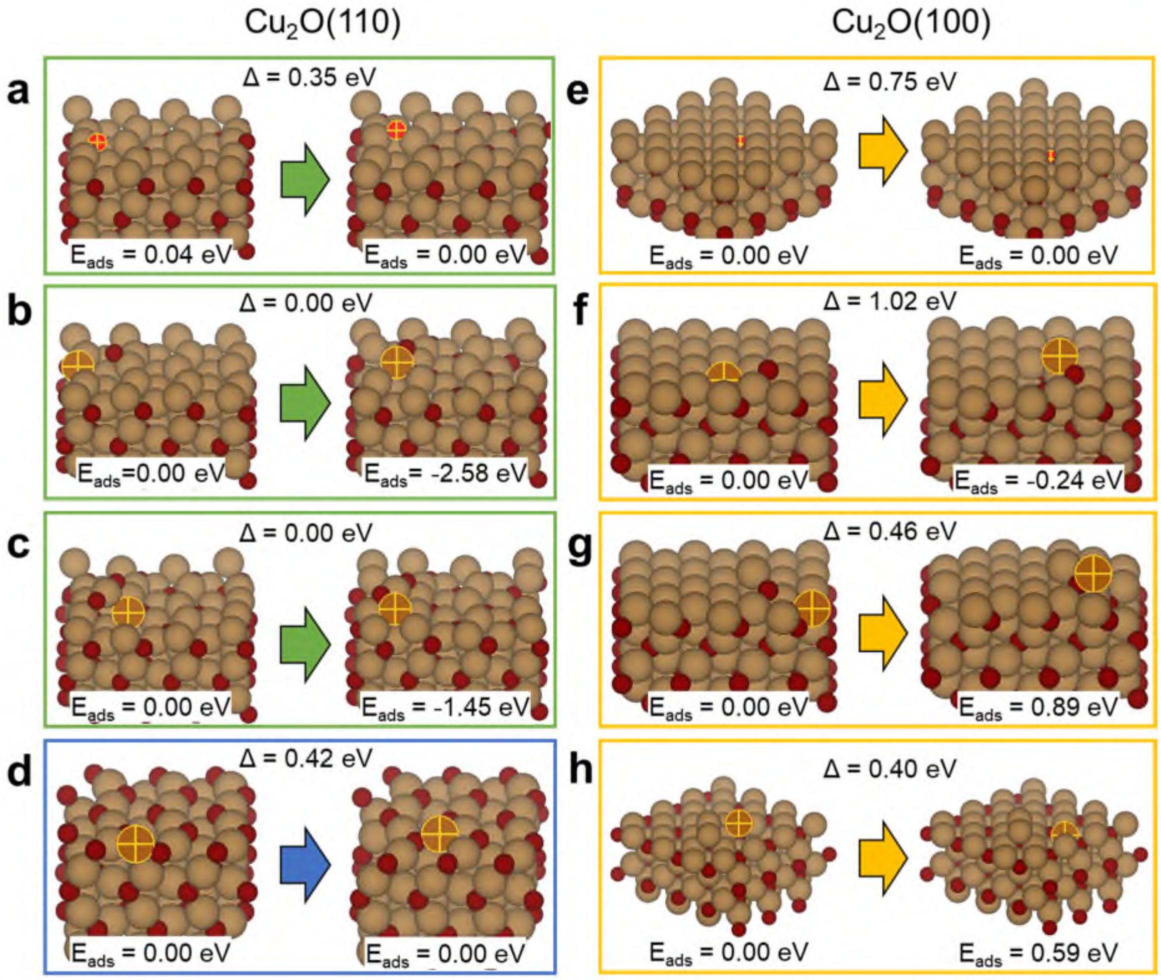

Supplementary Figure 31 | Summary of most favorable Cu and O diffusion processes on Cu_2O surfaces during oxidation, indicating adsorption (E_{ads}) and diffusion (Δ) energies for each process.

(**a-c**) Events for the growth of a new Cu-O terminated Cu_2O(110) layer on Cu terminated Cu_2O(110) surfaces by (**a**) first O diffusion, (**b**) first Cu diffusion, and (**c**) second Cu diffusion. (**d**) The single unique Cu diffusion process needed to grow a Cu-terminated layer on Cu-O terminated Cu_2O(110). (**e**) The most favorable O diffusion mechanism on Cu-terminated surfaces on Cu terminated Cu_2O(100). (**f-h**) The most favorable diffusion mechanisms depicting (**f**) first, (**g**) second, and (**h**) third Cu diffusion events with pre-existing O atoms. The diffusing atom is highlighted and marked with a cross in all figures.

Supplementary Table 6 | Summary of diffusion energetics of visualized structures.

Orientation	Adatom(s)	Event	Candidate diffusion event	E_{ads} (eV)	Δ (eV)
(100),Cu<br><br>Supplementary Figure 27	O	1	**a – subsurface diffusion**	**0.00**	**0.75**
			b – diffusion from Cu-O, Cu-O subsurface to Cu-O-Cu subsurface site	1.44	1.95
			c – diffusion from Cu-O, Cu-O subsurface to cross-channel site	1.03	1.33
			d – diffusion from Cu-O-Cu subsurface to cross-channel site	0.41	0.84
			e – cross-channel diffusion	0.00	1.47
(100),Cu<br><br>Supplementary Figure 28	Cu	1	1st Cu surface ejection to form first Cu-terminated layer	-0.24	1.02
	Cu	2	**2nd Cu ejection (adsorb diagonal to 1st Cu) to form first Cu-terminated layer**	**0.89**	**0.00**
			2nd Cu ejection (adsorb adjacent to 1st Cu) to form first Cu-terminated layer	0.98	0.00
	Cu	3	3rd Cu ejection to form first Cu-terminated layer	0.59	0.00
(110),Cu-O<br><br>Supplementary Figure 29	Cu	1	**a - in-channel diffusion**	**0.00**	**0.42**
			b - cross-channel diffusion on matching sites	0.00	1.02
			c - cross-channel diffusion on opposing sites	0.00	1.58
(110),Cu<br><br>Supplementary Figure 30	O	1	**a – subsurface to surface diffusion**	**0.00**	**0.35**
			b – in-channel diffusion	0.00	1.38
			c – cross-channel diffusion over adjacent (nearest possible) sites	0.00	1.16
			d – cross-channel diffusion over diagonal (next nearest possible) sites	0.00	1.98
	Cu	2	a – nearest neighbor diffusion	-1.78	-
			**b – second nearest neighbor diffusion**	**-2.58**	**-**
			c - third nearest neighbor diffusion	-1.72	0.40
	Cu	4	a – concerted diffusion of 2 Cu adatoms	-1.45	0.60
			b – in-channel diffusion	-1.43	-

Note that all adsorption (E_{ads}) and diffusion barrier (Δ) energies are normalized with respect to the lowest adsorption energy of each path that is 0 or greater, while listed E_{ads} values indicate either the higher (if greater than 0) or lower (if less than 0) adsorption energy in paths featuring more than one unique site (they are equal to zero if only one unique site is present).

Supplementary Note 8: Evaluating the effect of surface reconstruction on oxide growth

As shown in Supplementary Figure 9, surface reconstruction is observed on the Cu surface during oxidation.

In this work, we observed the missing row surface reconstruction on Cu(100) surface, as shown in Figure 3 and Supplementary Figure 8. This result is consistent with previous theoretical predictions and STM experiments[41, 42]. Although surface reconstruction has been speculated as an important transient stage in oxidation, the necessity of surface reconstruction on Cu_2O growth is still controversial, since recent experiments also reported oxide growth without surface reconstruction[43].

Nevertheless, since the focus of this work is on the growth of Cu_2O, the reconstruction states on the Cu surface would only affect the diffusion of Cu and O on Cu. To evaluate the effect of the missing row reconstructed Cu(100) surface on the Cu and O diffusion, the energetics involved in the Cu_2O growth process are compared as follows:

1. For O_2 dissociation, the dissociative adsorption of oxygen is blocked by the on-surface oxygen on the MRR reconstructed Cu surface[44]. In comparison, on Cu_2O (100) and (110) surfaces, O_2 dissociative adsorption is preferred over O_2 molecular adsorption[45]. Hence, the O source for the Cu_2O growth comes from the O atoms dissociated from O_2 on the Cu_2O surface.

2. For O diffusion, as shown in Supplementary Table 7, the diffusion barrier on Cu-terminated Cu_2O(100) and (110) surfaces calculated in this work (0.75 eV and 0.35 eV) are both much lower than that on the MRR Cu(100) surface (1.4 eV)[46] and on the clean Cu(100) surface (0.74 eV)[47]. Hence, even if there are O atoms (ionized by the electron beam) on the (reconstructed) Cu surfaces, O diffusion toward Cu_2O(110) surface is still preferred.

3. For Cu diffusion, as shown in Supplementary Table 7, the formation of missing row reconstruction has greatly increased the diffusion barrier of Cu from ~0.53 eV on clean Cu[47] to 2.0 eV on MRR Cu[46]. In comparison, the Cu diffusion barrier on Cu_2O(110) calculated in this work is only 0.42 eV. Hence, Cu diffusion toward Cu_2O(110) is also preferred. Besides, the Cu diffusion barrier in bulk Cu (~2.1 eV)[48] is only slightly higher than that of MRR Cu. This could explain the observed surface-to-bulk Cu source transition presented in Figure 3 at a relatively small distance range.

Although the surface reconstruction of Cu changes the surface diffusion energies on Cu, this work is focused on the growth of Cu_2O on Cu_2O islands, instead of the initial nucleation of Cu_2O from Cu. Computation of diffusion energies is carried out on Cu_2O surfaces, instead of Cu surfaces, in this work. To gain a comprehensive understanding of Cu_2O surface diffusion energetics, Cu and O diffusion on Cu_2O surfaces with both O-rich (O-terminated Cu_2O(100) and Cu-O terminated Cu_2O(110)) and O-lean (Cu-terminated Cu_2O(100) and (110)) terminations

were studied. Hence, the surface reconstruction of Cu would not affect evaluated DFT results, as such results reviewed diffusion energies on Cu_2O.

Supplementary Table 7| Summary of Cu and O diffusion barriers on different Cu and Cu_2O surface configurations

Surface	Diffusing atom	Activation energy (eV)	Ref.
**Clean Cu(100)**	O	0.74	[47]
	Cu	0.53	[47]
**MRR Cu(100)**	O	1.4	[46]
	Cu	2.0	[46]
**Bulk Cu**	Cu	2.1	[48]
**Bulk Cu_2O**	Cu	1.23	[49]
**$Cu_2O(100)$ -Cu terminated**	O	0.75	This work
**$Cu_2O(110)$ -Cu terminated**	O	0.35	
**$Cu_2O(110)$ - Cu-O terminated**	Cu	0.42	

Supplementary References

1. M L, C.L B, Scholz R. Evolution of hole size and shape in {100} {110} and {111} monocrystalline thin films of gold. *Thin Solid Films* **150**, 323-335 (1987).

2. Lowe DG. Distinctive image features from scale-invariant keypoints. *International journal of computer vision* **60**, 91-110 (2004).

3. Kresse G, Furthmüller J. Efficient iterative schemes for ab initio total-energy calculations using a plane-wave basis set. *Physical Review B* **54**, 11169-11186 (1996).

4. Kresse G, Joubert D. From ultrasoft pseudopotentials to the projector augmented-wave method. *Physical Review B* **59**, 1758-1775 (1999).

5. Perdew JP, Burke K, Ernzerhof M. Generalized gradient approximation made simple. *Physical Review Letters* **77**, 3865-3868 (1996).

6. Blöchl PE. Projector augmented-wave method. *Physical Review B* **50**, 17953-17979 (1994).

7. Bendavid LI, Carter EA. First-Principles Predictions of the Structure, Stability, and Photocatalytic Potential of Cu2O Surfaces. *The Journal of Physical Chemistry B* **117**, 15750-15760 (2013).

8. Bendavid LI, Carter EA. CO2 Adsorption on Cu2O(111): A DFT+U and DFT-D Study. *The Journal of Physical Chemistry C* **117**, 26048-26059 (2013).

9. Chi H, *et al.* Dependence of H2 and CO2 selectivity on Cu oxidation state during partial oxidation of methanol on Cu/ZnO. *Applied Catalysis A: General* **556**, 64-72 (2018).

10. Cococcioni M, de Gironcoli S. Linear response approach to the calculation of the effective interaction parameters in the $\mathrm{LDA}+\mathrm{U}$ method. *Physical Review B* **71**, 035105 (2005).

11. Curnan MT, Kitchin JR. Effects of concentration, crystal structure, magnetism, and electronic structure method on first-principles oxygen vacancy formation energy trends in perovskites. *The Journal of Physical Chemistry C* **118**, 28776-28790 (2014).

12. Dudarev SL, Botton GA, Savrasov SY, Humphreys CJ, Sutton AP. Electron-energy-loss spectra and the structural stability of nickel oxide: An LSDA+U study. *Physical Review B* **57**, 1505-1509 (1998).

13. Monkhorst HJ, Pack JD. Special points for Brillouin-zone integrations. *Physical Review B* **13**, 5188-5192 (1976).

14. Henkelman G, Jónsson H. Improved tangent estimate in the nudged elastic band method for finding minimum energy paths and saddle points. *The Journal of Chemical Physics* **113**, 9978-9985 (2000).

15. Henkelman G, Uberuaga BP, Jónsson H. A climbing image nudged elastic band method for finding saddle points and minimum energy paths. *The Journal of Chemical Physics* **113**, 9901-9904 (2000).

16. Saal JE, Kirklin S, Aykol M, Meredig B, Wolverton C. Materials design and discovery with high-throughput density functional theory: the open quantum materials database (OQMD). *Jom* **65**, 1501-1509 (2013).

17. Saidi WA, Lee M, Li L, Zhou G, McGaughey AJH. Ab initio atomistic thermodynamics study of the early stages of Cu(100) oxidation. *Physical Review B* **86**, 245429 (2012).

18. Dong Z, Zhang L, Wang S, Luo L. Direct visualization of dynamic atomistic processes of Cu2O crystal growth through gas-solid reaction. *Nano Energy* **70**, 104527-104527 (2020).

19. Hua Q, *et al.* Crystal-plane-controlled selectivity of Cu2O catalysts in propylene oxidation with molecular oxygen. *Angewandte Chemie - International Edition* **53**, 4856-4861 (2014).

20. Hua Q, *et al.* Morphological evolution of Cu2O nanocrystals in an acid solution: Stability of different crystal planes. *Langmuir* **27**, 665-671 (2011).

21. Soon A, Todorova M, Delley B, Stampfl C. Thermodynamic stability and structure of copper oxide surfaces: A first-principles investigation. *Physical Review B* **75**, 125420 (2007).

22. Lyubinetsky I, Lea AS, Thevuthasan S, Baer DR. Formation of epitaxial oxide nanodots on oxide substrate: Cu2O on SrTiO3(1 0 0). *Surface Science* **589**, 120-128 (2005).

23. Markworth PR, Liu X, Dai JY, Fan W, Marks TJ, Chang RPH. Coherent island formation of Cu2O films grown by chemical vapor deposition on MgO(110). *Journal of Materials Research* **16**, 2408-2414 (2001).

24. Ottosson M, Lu J, Carlsson JO. Chemical vapour deposition of Cu2O on MgO(100) from

CuI and N2O: aspects of epitaxy. *Journal of Crystal Growth* **151**, 305-311 (1995).

25. Egerton RF, Li P, Malac M. Radiation damage in the TEM and SEM. *Micron* **35**, 399-409 (2004).

26. Hansen TW, Wagner JB. *Controlled Atmosphere Transmission Electron Microscopy: Principles and Practice* (2016).

27. Sheng H, *et al.* Atomistic manipulation of reversible oxidation and reduction in Ag with an electron beam. *Nanoscale* **11**, 10756-10762 (2019).

28. Song M, *et al.* Oriented attachment induces fivefold twins by forming and decomposing high-energy grain boundaries. *Science* **367**, 40-45 (2020).

29. Zheng H, *et al.* Direct atomic-scale observation of layer-by-layer oxide growth during magnesium oxidation. *Applied Physics Letters* **104**, 141906-141906 (2014).

30. Yang JC, Yeadon M, Kolasa B, Gibson JM. The Limited Role of Surface Defects as Nucleation Sites for Cu2O on Cu (001). *Journal of the Electrochemical Society* **146**, 2103-2106 (1999).

31. Doudevski I, Hayes WA, Schwartz DK. Submonolayer island nucleation and growth kinetics during self-assembled monolayer formation. *Physical Review Letters* **81**, 4927-4930 (1998).

32. Tang L-H. Island formation in submonolayer epitaxy. *Journal de Physique I* **3**, 935-950 (1993).

33. Eberhardt A, Fenter P, Eisenberger P. Growth kinetics in self-assembling monolayers: A unique adsorption mechanism. *Surface Science* **397**, (1998).

34. Chow GC. Tests of equality between sets of coefficients in two linear regressions. *Econometrica: Journal of the Econometric Society*, 591-605 (1960).

35. Andrews DW. Tests for parameter instability and structural change with unknown change point. *Econometrica: Journal of the Econometric Society*, 821-856 (1993).

36. Zeileis A, Leisch F, Hornik K, Kleiber C. strucchange. An R package for testing for structural change in linear regression models. (2001).

37. Bartelt MC, Evans JW. Scaling analysis of diffusion-mediated island growth in surface adsorption processes. *Physical Review B* **46**, 12675-12687 (1992).

38. Shapiro SS, Wilk MB. An analysis of variance test for normality (complete samples). *Biometrika* **52**, 591-611 (1965).

39. Hothorn T, Zeileis A, Farebrother RW, Cummins C, Millo G, Mitchell D. lmtest: Testing linear regression models. *R package version 09-34, URL https://cran r-project org/package= lmtest,* (2015).

40. Momma K, Izumi F. VESTA 3 for three-dimensional visualization of crystal, volumetric and morphology data. *Journal of Applied Crystallography* **44**, 1272-1276 (2011).

41. Saidi WA, Lee M, Li L, Zhou G, McGaughey AJHH. Ab initio atomistic thermodynamics study of the early stages of Cu(100) oxidation. *Physical Review B - Condensed Matter and Materials Physics* **86**, 1-8 (2012).

42. Lahtonen K, Hirsimäki M, Lampimäki M, Valden M. Oxygen adsorption-induced nanostructures and island formation on Cu{100}: Bridging the gap between the formation of surface confined oxygen chemisorption layer and oxide formation. *Journal of Chemical Physics* **129**, (2008).

43. Zhou G, Luo L, Li L, Ciston J, Stach EA, Yang JC. Step-edge-induced oxide growth during the oxidation of Cu surfaces. *Physical Review Letters* **109**, 1-5 (2012).

44. Gattinoni C, Michaelides A. Atomistic details of oxide surfaces and surface oxidation: the example of copper and its oxides. *Surface Science Reports* **70**, 424-447 (2015).

45. Yu X, Zhang X, Tian X, Wang S, Feng G. Density functional theory calculations on oxygen adsorption on the Cu 2 O surfaces. *Applied Surface Science* **324**, 53-60 (2015).

46. Jaatinen S*, et al*. Adsorption and diffusion dynamics of atomic and molecular oxygen on reconstructed Cu(100). *Physical Review B - Condensed Matter and Materials Physics* **75**, 075402-075402 (2007).

47. Alatalo M, Jaatinen S, Salo P, Laasonen K. Oxygen adsorption on Cu(100): First-principles pseudopotential calculations. *Physical Review B - Condensed Matter and Materials Physics* **70**, 1-6 (2004).

48. Tan CM, Roy A. Electromigration in ULSI interconnects. *Materials Science and Engineering R: Reports* **58**, 1-75 (2007).

49. Peterson NL, Wiley CL. Diffusion and point defects in Cu2O. *Journal of Physics and Chemistry of Solids* **45**, 281-294 (1984).
