# Supplementary material for: Unusual layer-by-layer growth of epitaxial oxide islands during Cu oxidation
Source: Nat Commun. 2021 May 13;12:2781. doi: 10.1038/s41467-021-23043-w (PMC8119701; doi:10.1038/s41467-021-23043-w)
Supplement: Supplementary file 3 — Description of Additional Supplementary Files [file 41467_2021_23043_MOESM3_ESM.pdf]

Description of Additional Supplementary Files

Supplementary Movie 1

a *In situ* ETEM observation of layer-by-layer growth of Cu_2O along Cu_2O(110) during oxidation of Cu(100) facet at 300 °C under 0.3 Pa O_2. **b** Enlarged view of the boxed area in **(a)** and the corresponding schematic showing the identified grown layers for measured data plotted in **(c-d)**. **c** Measured growth trajectories of two ends of each layer with time. **d** Measured projection length of each layer with time.

Supplementary Movie 2

Layer-by-layer growth of Cu_2O along Cu_2O(110) on Cu(110) facet and Cu(100) facet during *in situ* ETEM oxidation at 300 °C under 0.3 Pa O_2.

Supplementary Movie 3

Layer-by-layer growth of a Cu_2O island growing on Cu(001) terrace at 300 °C under 0.15 Pa O_2.

Supplementary Movie 4

Layer-by-layer growth of Cu_2O along Cu_2O(110) in later stage oxidation when large oxide island is formed at 300 °C under 0.3 Pa O_2.

Supplementary Movie 5

In situ ETEM observation of Cu source for Cu_2O growth changed from step edges to the interface when the distance between Cu_2O island and nearby Cu step edge increases. The Cu||Cu_2O interface at t=0 is marked by the dashed line.
